# Mechanisms of ferroptotic and non-ferroptotic organ toxicity of chemotherapy: protective and therapeutic effects of ginger, 6-gingerol and zingerone in preclinical studies

**DOI:** 10.1007/s00210-024-03623-5

**Published:** 2024-12-05

**Authors:** Ademola C. Famurewa, Roland E. Akhigbe, Mina Y. George, Yemi A. Adekunle, Precious A. Oyedokun, Tunmise M. Akhigbe, Amos A. Fatokun

**Affiliations:** 1 Department of Medical Biochemistry, Faculty of Basic Medical Sciences, College of Medical Sciences, Alex Ekwueme Federal University Ndufu-Alike, Ikwo, Nigeria; 2 Centre for Natural Products Discovery, School of Pharmacy and Biomolecular Sciences, Faculty of Science, Liverpool John Moores University, Byrom Street, Liverpool, L3 3AF UK; 3 Department of Physiology, Ladoke Akintola University of Technology, Ogbomoso, Oyo State Nigeria; 4 Reproductive Biology and Toxicology Research Laboratory, Oasis of Grace Hospital, Osogbo, Nigeria; 5 Department of Pharmacology and Toxicology, Faculty of Pharmacy, Ain Shams University, Cairo, 11566 Egypt; 6 Department of Pharmaceutical and Medicinal Chemistry, College of Pharmacy, Afe Babalola University, Ado-Ekiti, Nigeria; 7 Breeding and Genetics Unit, Department of Agronomy, Osun State University, Osogbo, Osun State Nigeria

**Keywords:** Ferroptosis, Chemotherapy, Anticancer drugs, Ginger rhizome, *Zingiber officinale*

## Abstract

Chemotherapy (CT) is one of the flagship options for the treatment of cancers worldwide. It involves the use of cytotoxic anticancer agents to kill or inhibit the proliferation of cancer cells. However, despite its clinical efficacy, CT triggers side effect toxicities in several organs, which may impact cancer patient’s quality of life and treatment outcomes. While the side effect toxicity is consistent with non-ferroptotic mechanisms involving oxidative stress, inflammation, mitochondrial impairment and other aberrant signalling leading to apoptosis and necroptosis, recent studies show that ferroptosis, a non-apoptotic, iron-dependent cell death pathway, is also involved in the pathophysiology of CT organ toxicity. CT provokes organ ferroptosis via system Xc^–^/GPX-4/GSH/SLC7A11 axis depletion, ferritinophagy, iron overload, lipid peroxidation and upregulation of ferritin-related proteins. Cisplatin (CP) and doxorubicin (DOX) are common CT drugs indicated to induce ferroptosis in vitro and in vivo. Studies have explored natural preventive and therapeutic strategies using ginger rhizome and its major bioactive compounds, 6-gingerol (6G) and zingerone (ZG), to combat mechanisms of CT side effect toxicity. Ginger extract, 6G and ZG mitigate non-ferroptotic oxidative inflammation, apoptosis and mitochondrial dysfunction mechanisms of CT side effect toxicity, but their effects on CT-induced ferroptosis remain unclear. Systematic investigations are, therefore, needed to unfold the roles of ginger, 6G and ZG on ferroptosis involved in CT side effect toxicity, as they are potential natural agents for the prevention of CT toxicity. This review reveals the ferroptotic and non-ferroptotic toxicity mechanisms of CT and the protective mechanisms of ginger, 6G and ZG against CT-induced, ferroptotic and non-ferroptotic organ toxicities.

## Introduction

Cancer treatment has made considerable strides over the past 60 years due to advancements in various therapeutic options and strategies. Although the traditional treatment options are still surgery, radiotherapy and chemotherapy, the landscape of tumour treatment has largely changed comprehensively and remarkably, ushering in cutting-edge therapeutic strategies (Liu et al. 2024). The emerging strategies have not only afforded personalised and precise tumour targeting, but also provided patients with enhanced therapeutic comfort, the potential to impede disease progression and an increased prospect of surviving cancer (based on the number of cancer survivors) (Lomeli et al. 2021; Liu et al. 2024). Targeted therapy, cell therapy (e.g. CAR-T cell therapy), gene therapy (e.g. mRNA therapy), monoclonal antibody therapy, antibody drug conjugates, small molecule inhibitors, proton therapy and carbon ion therapy, neoantigen/cancer vaccines and oncolytic virus, and photothermal and photodynamic therapy have been approved and introduced to clinical oncotherapy (Thurston 2007; Pucci et al. 2019; Liu et al. 2024). Thermal ablation of tumours and magnetic hyperthermia are opening new opportunities for precision medicine, making the treatment localised to very narrow and precise areas (Pucci et al. 2019). Taken together, these strategies will be able to provide the best personalised therapies for cancer patients, highlighting the importance of combining multiple disciplines to get the best outcomes. However, these treatments are still associated with contending challenges and drawbacks, including cytokine toxicities, adverse immune responses and off-target tissue damage. The treatment often encompasses more than one approach, and the strategy to be adopted largely depends on the type, nature and progression of the cancer. The traditional treatment options, radiotherapy and chemotherapy, generate significant side effects in patients, linked to their anticancer mechanisms, doses and frequency of use towards achieving remission.

Systemic and cytotoxic chemotherapy (CT) is still the frontline treatment modality for various cancers. Coined by Paul Ehrlich, a German chemist, CT is the use of cytotoxic chemical drugs to kill or inhibit the proliferation of cancer cells, and its anticancer efficacy has increased the number of cancer survivors (Lomeli et al. 2021). Due to its effectiveness in killing rapidly dividing cells, a prominent cancer hallmark, CT remains a cornerstone of cancer treatment thus far despite the introduction of newer targeted and gene/antibody-based therapies (Brianna and Lee 2023). The cytotoxic CT agents target inhibition of tubulin dynamics, DNA replication and transcription, and metabolite synthesis in cancer cells. The blockage of the mitotic and metabolic processes in cancer cells via various molecular mechanisms leads to apoptotic and non-apoptotic cancer cell death (Al-Amir et al. 2023; Wang et al. 2024a). Despite the indisputable potency of CT in oncological treatments, both clinical and experimental data have strongly indicated that CT agents, such as tamoxifen (TAM), cisplatin (CP), cyclophosphamide (CYP), doxorubicin (DOX), methotrexate (MTX), 5-fluorouracil (5-FU), paclitaxel (PXT) and docetaxel (DXT), dissipate severe side effect toxicity on various organs of the body (Famurewa et al. 2022; Wang et al. 2024a). In other words, the anticancer mechanisms of CTs exert deleterious effects on healthy cells and organs, thereby orchestrating side effect toxic damage on the liver, kidney, brain, spinal cord, testis, ovary, placenta, heart, lungs, cochlea and spleen. However, while abundant pieces of evidence indicate that non-ferroptotic toxicity mechanisms, including oxidative stress, inflammation, membrane dysfunction, apoptosis, pyroptosis and other signalling pathways, are critically involved in CT-induced organ toxicity (Okkay et al. 2024; Zhou et al. 2023; Famurewa et al. 2023; Fang et al. 2023; Nagoor Meeran et al. 2023), interestingly, recently published literature has shown that the ferroptotic pathway may also be involved in CT-induced organ toxicity. Ferroptosis is a recently discovered, non-apoptotic, programmed cell death pathway provoked by iron-mediated lipid peroxidation and ROS through impairment of iron metabolism and glutathione-glutathione peroxidase 4 (GSH-GPX4) signal transduction pathways (Wang and Yi 2024; Zhao et al. 2023). It plays a pivotal role in several pathophysiological conditions, including diabetes, neurodegenerative disorders, ischemia–reperfusion injury and carcinogenesis (Li et al. 2022). CT-induced iron overload and altered iron metabolism leading to ferroptosis and tissue damage in certain organs have been reported in recent studies (Jian et al. 2021; Ikeda et al. 2021). Interestingly, the molecular cues involved in ferroptosis-mediated CT toxicity suggest that natural products with potent pharmacological activities may elicit anti-ferroptosis activity if used in the treatment of CT-induced toxicity.

**Fig. 1:**
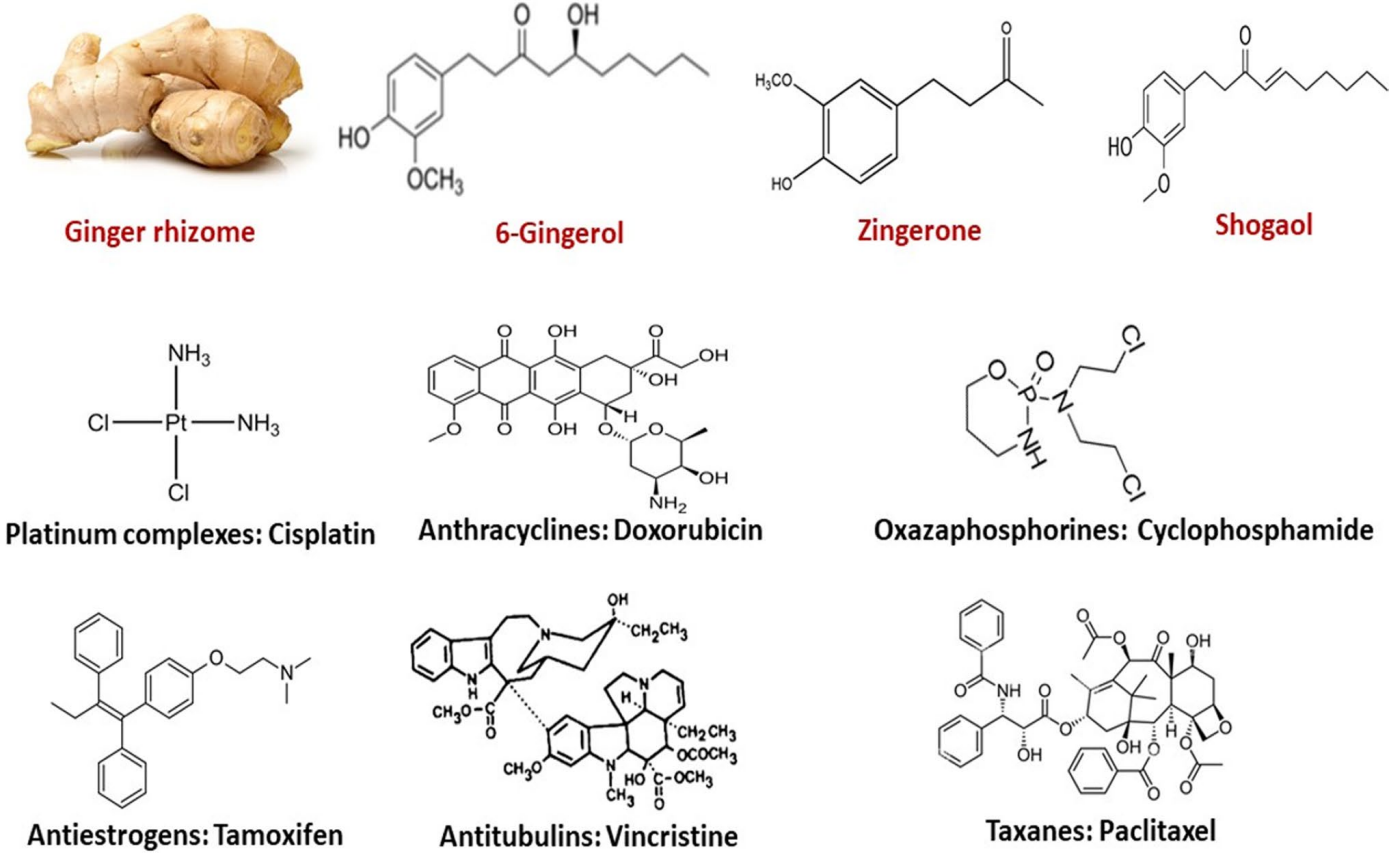
Ginger rhizome, the molecular structures of its most bioactive compounds and common examples of CT drugs from the different classes of cytotoxic anticancer drugs

Research in natural products and screening of plant-derived products are ongoing towards discovering natural agents for combating CT-induced organ toxicity. Ginger (*Zingiber officinale*) is a natural herb with a longstanding medicinal background in treating various disorders and diseases (Xiang et al. 2024). It is commonly used as a spice in various foods and beverages around the world, and as a dietary supplement in traditional remedies. Its remarkable biological activities are well known, associated with ginger solvent extract and its various bioactive compounds. Ginger and its components have shown antioxidant, anti-inflammatory, antiapoptotic and immunomodulatory effects, as well as modulatory effects on signalling pathways and prevention of toxicity and pathologies in in vitro and in vivo models (Ayustaningwarno et al. 2024). Ginger’s antioxidant and anti-inflammatory activities are ascribed to gingerol, zingerone, shogaols and paradol. The main component of ginger is gingerol, which is principally responsible for the pungent and spicy taste of fresh ginger rhizome (Wohlmuth et al. 2005). Moreover, a robust body of studies have shown that ginger extracts and its foremost components, 6G and ZG (Fig. 1), possess protective activities against CT-induced organ toxicity. Therefore, the aim of this review was to summarize the recent investigations revealing the mechanistic roles of ferroptosis and non-ferroptosis pathways in CT side effect toxicity in preclinical studies, with an overall emphasis on the inhibition of these pathways by ginger rhizome extract, 6G and ZG, as reported in the preclinical literature. We searched relevant databases, including Web of Science, Scopus, PubMed and Google Scholar, for published papers on CT-induced ferroptosis-mediated toxicity; the mitigation of CT toxicity via targeting ferroptosis and non-ferroptosis pathways by ginger rhizome extract, 6G and ZG; and the protective mechanisms of these natural products against CT-induced organ toxicity. This review will further promote a comprehensive understanding of the molecular pathways with respect to ferroptosis modulation that may be responsible for the protection and therapeutic effects of ginger rhizome extract, 6G and ZG as an adjuvant therapy alongside CT treatments. The significance of these insights lies in the recognition of the involvement of the targetable cell death process of ferroptosis, the modulation of which holds promise for enhancing the quality of life for patients during and after treatment.

### Anticancer drugs

Cancer is a disease in which the control of growth signalling is dysregulated in one or more cells, leading to an uncontrolled cell proliferation and growth of a mass of cells known as a tumour. The clinical use of cytotoxic anticancer agents for the treatment of cancers is termed CT. Although there are other established therapeutic options, including surgery and radiotherapy, CT retains a distinction as a treatment modality due to its effectiveness in killing rapidly dividing cells and/or metastasized cancer cells that are a prominent hallmark of cancer (Brianna and Lee 2023). Notably, CT has extended the survival of innumerable cancer patients. A German chemist, Paul Ehrlich, in the early 1900s coined the name ‘chemotherapy’ because it involves the use of cytotoxic chemical agents that target different stages of mitotic and metabolic processes in order to inhibit the cell cycle, cell division and growth of cancer cells. (Kaufmann 2008). The classes of cytotoxic anticancer drugs (Fig. 1) used in CT treatment include antimetabolites (e.g. MTX and 5-FU), oxazaphosphorines (e.g. CYP), platinum complexes (e.g. CP), anthracyclines (e.g. DOX), antitubulins (e.g. vincristine and vinblastine), taxanes (e.g. PTX and DTX), topoisomerase inhibitors (e.g. topotecan and etoposide), anti-estrogens (e.g. TAM, toremifene) (Fig. 1) and a host of cell cycle inhibitors, anti-androgens and kinase inhibitors. CT can be used as a primary regimen for treating cancer, as a neoadjuvant before the application of a primary treatment regimen, or as an adjuvant to kill the remnant of cancer cells after the primary treatment (Seufferlein et al. 2014). They are administered through various routes, such as intravenous, oral, subcutaneous, intraventricular or intramuscular. The choice of route is based on considering the drugs’ pharmacokinetic properties, convenience of administration and patient’s health status (Brianna and Lee 2023; Aisner 2007). In clinical practice, some of the CTs are used as combination dosage regimens, which has been reported to have higher efficacy than monotherapy. The combination regimens exert synergistic or additive anticancer effects compared to monotherapy and also reduce the chances of chemoresistance and relapses via targeting the cancer stem cells. The anticancer mechanisms of various classes of drugs are mainly via DNA interactions. Majority of cytotoxic anticancer drugs exert cytotoxic actions by interacting with the DNA machinery of cancer cells to inhibit replication, transcription, translation and cell division (Brinkmann and Fritz 2022). The cytotoxic anticancer drug–induced DNA impairment triggers mechanistic signalling that culminates in inhibition of DNA repair mechanisms, cell-cycle arrest, ROS generation, mitochondrial dysfunction, endoplasmic reticulum (ER) damage and intrinsic and extrinsic apoptotic cell death (Hilton et al. 2015). However, the intractable drawbacks associated with CTs are chemoresistance and side effect toxicity (Davodabadi et al. 2023).

### Side effect organ toxicity of cytotoxic CT drugs

**Fig. 2:**
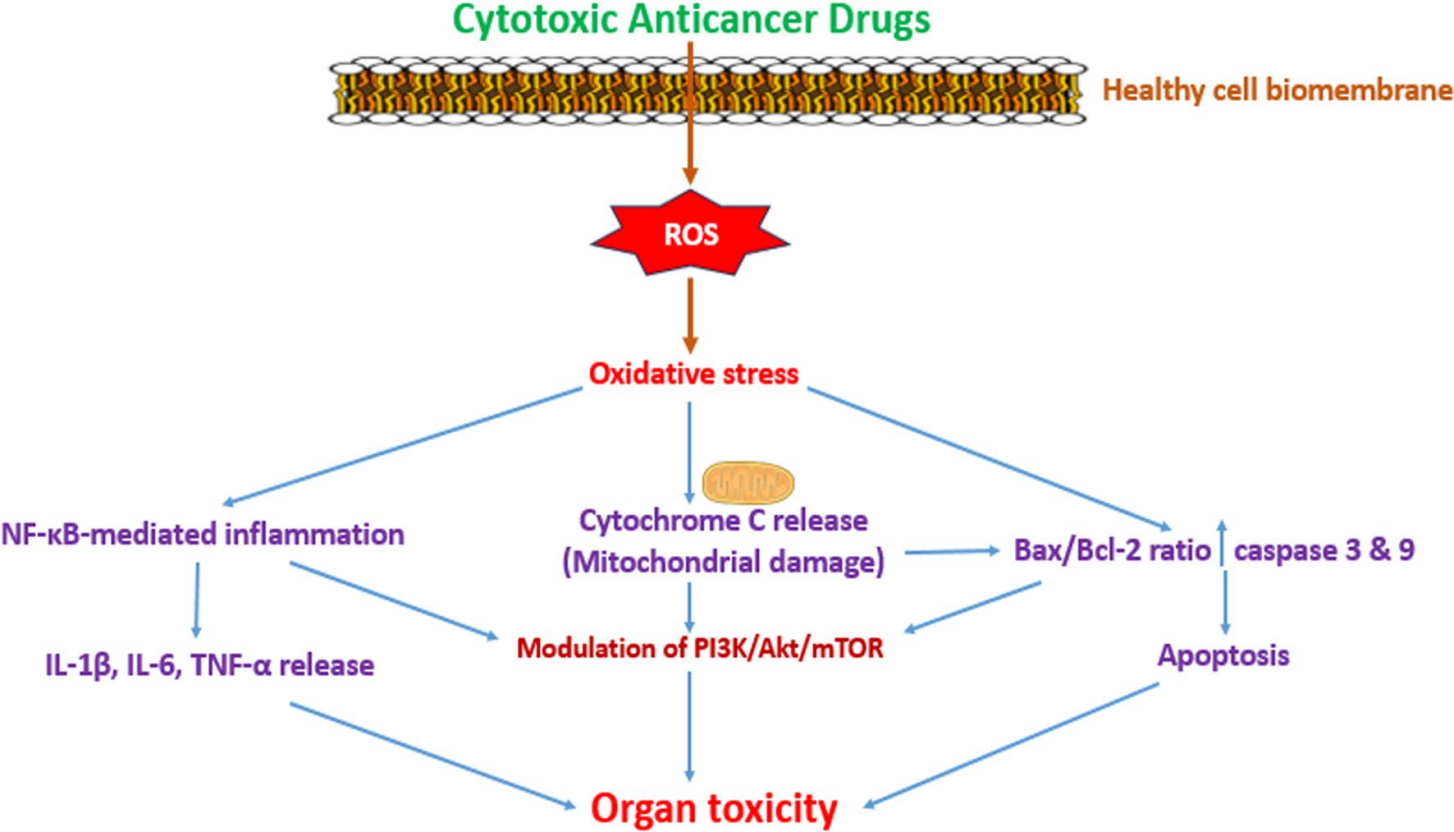
Mechanisms underlying chemotherapeutic drug-induced organ toxicity. Cytotoxic anticancer drugs generate ROS and/or oxidative stress. Oxidative stress triggers activation of NF-kB-mediated inflammation, mitochondrial impairment and intrinsic caspase-dependent apoptosis, leading to organ damage or toxicity

Besides chemoresistance, side effect organ toxicity is a prevailing and intractable drawback of CT. CTs not only target the cancer cells but also exert toxic effects on healthy cells in cancer patients. The non-targeted adverse effects affect major organs, leading to hepatotoxicity, ototoxicity, nephrotoxicity, cardiotoxicity, neurotoxicity, placental toxicity, testicular toxicity, lung toxicity, intestinal toxicity, ovarian toxicity and spleen toxicity (Famurewa et al. 2020a, b; Famurewa et al. 2017; Zhang et al. 2018b; Fang et al. 2023). For example, CYP, an alkylating oxazaphosphorine, is well known to induce bladder urotoxicity, clinically termed hemorrhagic cystitis (Famurewa et al. 2021; Zirak et al. 2020). DOX mainly targets the heart, causing cardiotoxicity; CP, a platinum complex, is associated with classic nephrotoxicity (Famurewa et al. 2022), while taxanes clinically induce peripheral neuropathy (Da Costa et al. 2020). It has been shown that the toxicity is orchestrated via ROS generation and subsequent development of oxidative stress. Oxidative stress in turn activates NF-κB-mediated inflammatory mechanism and caspase-dependent apoptosis cascades, leading to mitochondrial dysfunction and cell death (Nagoor Meeran et al. 2023; Famurewa et al. 2022). These impaired signalling networks mediate the role of PI3K/Akt/mTOR/GSK3/MAPK, endoplasmic reticulum stress mediators and autophagy in CT-induced organ toxicity (Nagoor Meeran et al. 2023; Jiang et al. 2023; Zhang et al. 2020) (Fig. 2). In addition, a number of studies have also implicated the mechanistic roles of microRNAs and long non-coding RNAs in CT-induced cardiotoxicity (Guan et al. 2023; Kawano and Adamcova 2022; Yu et al. 2022b). However, ferroptosis is an emerging, nonapoptotic form of cell death characterized by iron dependence and lipid peroxidation, which has been recently linked to CT-induced toxicity. Ferroptosis is involved in a range of pathologies, including cardiovascular diseases, neurodegeneration and cancer (Zhang et al. 2024; Wang et al. 2024b). Some systematic investigations have reported the crucial role of ferroptosis in CP-induced hearing loss ototoxicity (Jian et al. 2021), nephrotoxicity (Lin et al. 2024; Ikeda et al. 2021) and DOX-induced cardiotoxicity and nephrotoxicity (Ouyang et al. 2023; Tadokoro et al. 2020).

## Ferroptosis as a mechanism in chemotherapy-induced side effect toxicity

**Fig. 3:**
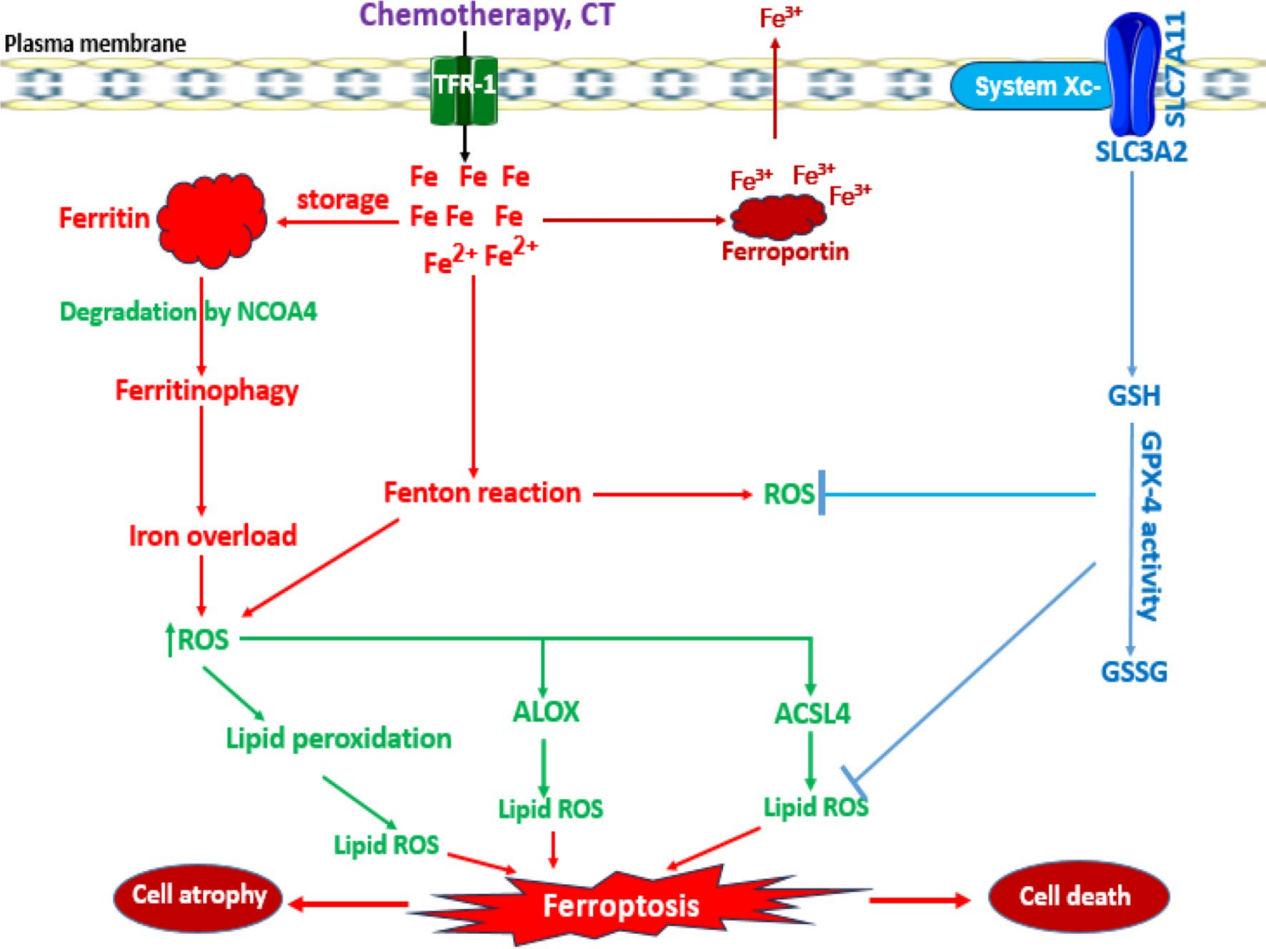
A schematic description of chemotherapy (CT)-induced ferroptosis. CT induces iron accumulation through transferrin receptor-1 (TFR-1) in cells, which leads to cellular iron overload. The binding of nuclear receptor coactivator-4 (NCOA4) to ferritin induces degradation of ferritin, a process known as ferritinophagy. As a result, cellular iron increases, causing iron overload–dependent excessive ROS accumulation, lipid peroxidation and ferroptosis. The cysteine/glutamate transporter receptor (Xc^−^) system (SLC3A2 + SLC7A11) enhances GSH synthesis and GPX-4 activity to inhibit ROS levels and lipid peroxidation. The activities of acyl-CoA synthetase long-chain family member 4 (ACSL4) and arachidonate lipoxygenase (ALOX) contribute to lipid peroxidation, ROS generation and ferroptosis

**Fig. 4:**
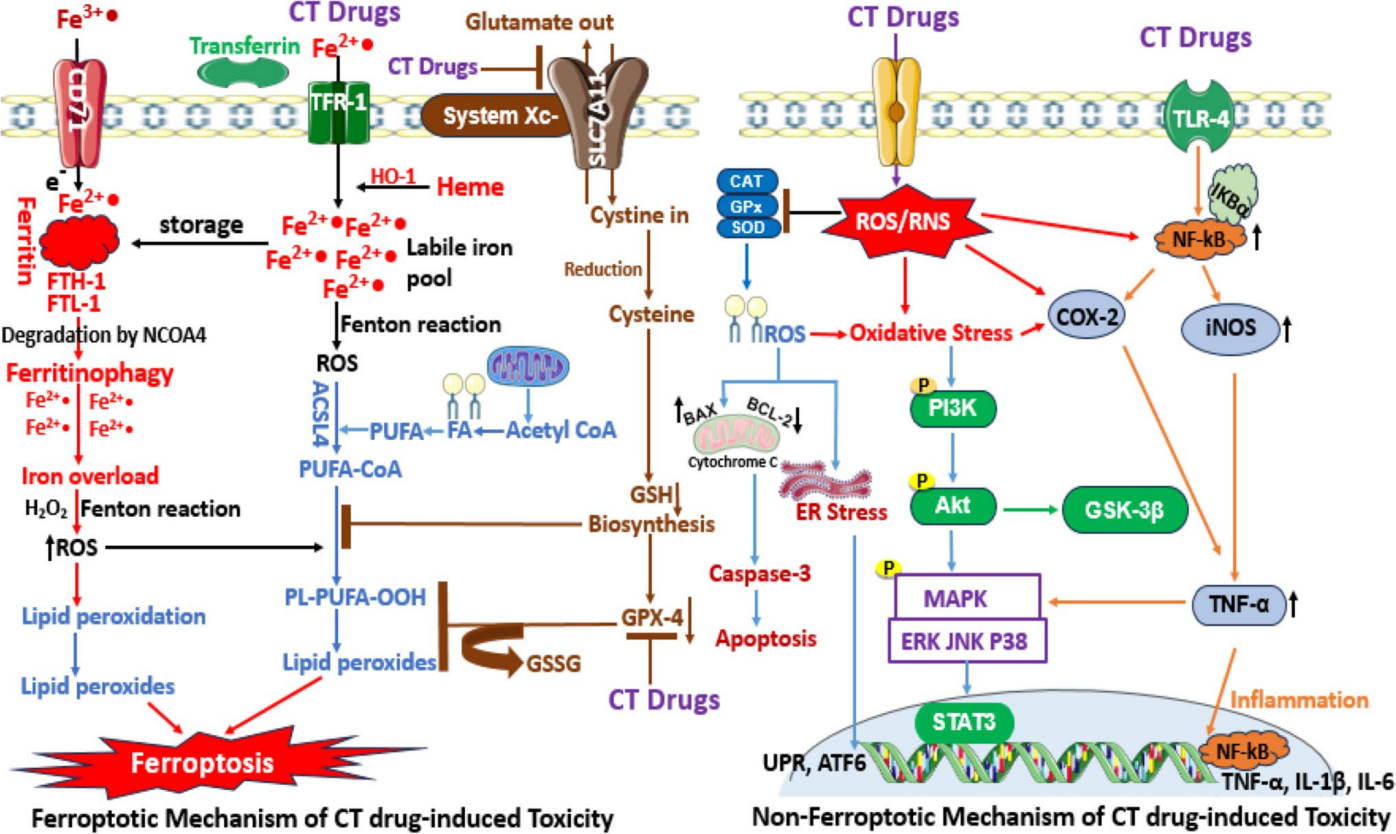
Schematic illustration of the ferroptotic and non-ferroptotic pathways triggered by cytotoxic chemotherapy drugs (CT drugs) in non-targeted, healthy cells. CT induces the entry of iron (II) via the transferrin and membrane protein transferrin receptor-1 (TFR-1). The increasing concentrations of iron (II) ions set up Fenton reactions and degradation of iron-storage protein ferritin by nuclear receptor coactivator-4 (NCOA4) through an autophagy-mediated process called ferritinophagy. These result in iron overload, increased ROS generation and formation of phospholipid-polyunsaturated fatty acid peroxides (PL-PUFA-OOH) by the action of acyl-CoA synthetase long-chain family member 4, ACSL4. Due to inhibition of the glutathione-glutathione peroxidase-4 (GSH-GPX-4) synthetic pathway by CT drugs, lipid peroxides are copiously generated, leading to cell death by ferroptosis. CT drugs initiate non-ferroptotic pathway through generation of ROS and activation of inflammatory processes. The build-up of oxidative stress triggers inhibition of antioxidant enzyme activities, mitochondrial dysfunction, ER stress, activation of MAPK-PI3K axis and NF-κB-mediated inflammatory cascades. These ROS-mediated alterations result in apoptosis and inflammation

Ferroptosis is a recently discovered programmed cell death induced by iron-dependent lipid peroxidation, ROS and oxidative damage. In 2012, ferroptosis was formally defined and recognised as a nonapoptotic cell death with hallmarks consistent with dysregulation of iron homeostasis and lipid peroxidation of polyunsaturated fatty acids in membrane phospholipids (Zhao et al. 2023). The programmed cell death expresses morphology characterized by reduction or absence of the mitochondrial crista, rupture of the outer membrane, decreased mitochondrial volume and increased membrane density (Baiskhanova and Schäfer 2024). It may be provoked via altered iron metabolism, iron overload and metabolism of amino acids and lipids. Although the elemental culprit is iron, iron is an essential micronutrient element and plays essential roles in metabolism, enzyme action and oxygen transport in red blood cells. However, excessive iron accumulation and/or iron overload may result from increased iron uptake via the transferrin receptor (CD71), which generates ferrous iron (Fe^2+^) stored in ferritin protein, with the association of ferritin heavy chain 1 (FTH-1) and ferritin light chain 1 (FTL-1). Ferritin iron storage capacity, which ranges from 2000 to 4500 Fe^2+^ ions, is enlarged due to accumulation resulting from transferrin and transferrin receptor-1 (TFR-1) intracellular transportation of iron. However, a lower expression of FTH-1 and FTL-1, coupled with transferrin receptor upregulation, can lead to excessive iron build-up, Fenton reactions and lipid peroxidation. The degradation of ferritin by the action of nuclear receptor coactivator 4 (NCOA4)–mediated autophagy, termed ferritinophagy, increases the level of labile iron pool or iron overload status, promoting Fenton reactions that generate iron (III) (Fe^3+^) and the hydroxyl radical (OH^·^), thus elevating ROS levels and inducing oxidative damage (Fig. 4). A key oxidative effect is lipid peroxidation via enhanced activity of acyl‑CoA synthetase long‑chain family member 4 (ACSL4), arachidonate lipoxygenase (ALOX) and its prolyl hydroxylase domain for the production of activated polyunsaturated fatty acid coenzyme A (PUFA-CoA) and phospholipid PUFA proxides in the presence of excess ROS (Chen et al. 2021b). In this oxidative milieu, several pathways are agitated, including cystine/glutathione/GPX4, ferroptosis suppressor protein-1/coenzyme Q10 (FSP1/CoQ10) and GTP cyclohydrolase-1/tetrahydrobiopterin/dihydrofolate reductase (GCH1/BH4/DHFR) signalling axis, activating downstream signalling pathways, inducing ferroptosis (Zhao et al. 2023; Hu et al. 2022a; Lee et al. 2020). The tripeptide antioxidant, glutathione (GSH), is a vital component of the cellular antioxidant defense and homeostasis. The process of its de novo synthesis occurs through the glutamate-cysteine transporter system called system Xc^−^, and the channel SLC7A11 which allows glutamate exit from the cell, and entry of cystine into the cell. Subsequently, cystine is converted to cysteine, which is further utilized in the synthesis of GSH. CT drugs have the potential to inhibit the function of system Xc^−^, which may lead to a reduction in GSH levels and activity of glutathione peroxidase-4 (GPX-4). Consequently, this may result in the accumulation of intracellular iron-generated ROS (Hu et al. 2020, 2022b). The GPX-4 enzyme plays a vital role in cellular antioxidant defense by converting lipid peroxides into lipid alcohols via the utilization of reduced GSH, thereby preventing the accumulation of lipid peroxides and subsequent damage to cell membranes and mitochondria, underscoring the role of SLC7A11/GPX-4 axis in the modulation of ferroptosis. Considering the importance of this enzyme for maintaining intracellular antioxidant balance (Famurewa et al. 2024), inhibition of SLC7A11/GPX-4 axis by CT drugs can directly lead to the initiation of ferroptosis (Ma et al. 2022). Therefore, in cells undergoing ferroptosis, GSH depletion, glutathione peroxidase-4 (GPX4) inactivation and accumulation of malondialdehyde, 4-hydroxynonenal, iron ions and lipid peroxides are the evident biochemical alterations (Baiskhanova and Schäfer 2024). GPX-4 is a master regulator and marker of ferroptosis; GPX-4’s decreased activity, followed by increased activity of ACSL4, leads to iron accumulation and induction of ferroptosis (He and Shi 2023). Oxidative impairment in the antioxidant cysteine/glutamate transporter receptor (Xc^−^) system causes a depletion of the synthesis of cysteine and GSH, a tripeptide antioxidant molecule (Wang et al. 2024a, b). GSH is used by GPX-4 to convert phospholipid hydroxylperoxide (PLOOH) to alcohol. The GSH and the system Xc^−^ play synergistic roles in enhancing detoxification of ROS, balance of cellular antioxidant mechanism and inhibiting oxidative stress to prevent ferroptosis (He and Shi 2023). Therefore, a reduced level of cysteine due to suppression of system Xc^−^ inactivates GPX-4 activity, ultimately leading to ferroptotic cascades (Wang et al. 2024b; Vera et al. 2024) (Figs. 3 and 4).

Ferroptosis as an emerging cell death mechanism has been indicated to be involved in the pathogenesis of CT-induced organ toxicity. In a recently published paper, exposure of auditory cochlear to CP caused intracellular free iron accumulation. The authors suggest that the iron accumulation may be linked to ferritin degradation, a type of autophagy named ferritinophagy due to increased expression of LC3II and decreased expression of NCOA4 as ferritinophagy biomarkers (Jian et al. 2021). In addition, levels of markers of ferroptosis, GSH, GPX-4 and solute carrier family 7 member 11 (SLC7A11), were reduced in the cochlear HEI-OC1 cell line following the elevated levels of lipid peroxidation markers. Studies have shown that CP decreased GPX-4 activity, GSH, SLC7A11 and system Xc^−^, and increased protein expression of ferritin, transferrin receptor-1 (TFR1), ACSL4, free iron level, ROS, 4-hydroxynonenal (4HNE) and malondialdehyde (MDA) in mouse kidney and in the renal HK-2 cell line (Song et al. 2024a, b; Li et al. 2023; Hu et al. 2020, 2022b; Zhou et al. 2022; Ikeda et al. 2021). In the Song et al. (2024a) study, CP induced reduced gene expression of ferritin heavy chain (FTH) and ferritin light chain (FTL). The study concludes that ferroptosis is involved in the pathogenesis of CP-induced renal toxicity. Interestingly, before the discovery of ferroptosis in 2012, Baliga et al. (1998) had reported evidence suggesting a role for iron in CP-induced nephrotoxicity. In the study, CP caused in vivo and in vitro (in LLC-PK1 cells, renal tubular epithelial cells) elevation of iron content accompanied with renal toxicity. This result was validated by the use of iron chelators (deferoxamine and 1,10-phenanthroline), which considerably reduced the CP-induced cytotoxicity. The HK-2 cells were employed to evaluate the underlying mechanisms of CP-induced renal kidney injury in a recent study (Lin et al. 2024). The study found that ferritinophagy is the key mechanism promoting iron overload and ferroptosis in CP-exposed HK-2 cells. According to the study (Lin et al. 2024), the ferroptotic cell death was confirmed by the reduced expression of GSH, GPX-4, ferritin heavy chain (FTH) and SQSTM1, while nuclear receptor coactivator4 (NCOA4) and LC3II/I were markedly upregulated. Mechanistically, therefore, CP could orchestrate ferroptosis via modulation of ferritinophagy-related genes and proteins. DOX is another efficacious CT for treatment of neoplastic diseases. However, it induces a classic form of cardiotoxicity, in which ferroptosis has been implicated. DOX-induced cardiotoxicity was mediated by ferroptosis via downregulation of antioxidant GPX-4 and SLC7A11 in the cardiomyocyte H9c2 cells (He et al. 2023). In that cell line, the effects of CP on genes for ferritin heavy chain 1 (FTH1), ferritin light chain 1 (FTL2), transferrin receptor-1 (TFR1, an iron importer protein) and ferroportin 1 (FPN1) were investigated. They are genes mainly responsible for regulating the intercellular iron homeostasis. The study found that DOX upregulated the gene expression of FTH1, FTL2 and TFR1, and downregulated the FPN1 gene to induce ferroptosis. DOX (Adriamycin) induces renal damage via oxidative stress, evidenced by iron content, ACSL4, ROS, MDA and depleted expression of SLC7A11 and activities of GPX-4, GSH and SOD (Ouyang et al. 2023). In DOX-induced cardiotoxicity, the levels of iron, ROS and lipid peroxidation MDA were elevated and that of GPX-4 depressed in mitochondria dysfunction-mediated ferroptosis in male C57BL/6 J mice (Tadokoro et al. 2020). Moreover, in vivo and in vitro studies have shown that downregulation of a lipid metabolism enzyme, acyl-CoA thioesterase 1 (Acot1), could contribute to DOX ferroptosis (Liu et al. 2020). The knockdown of Acot1 gene progresses the cardiomyocytes to ferroptosis in DOX-induced cardiotoxicity. Acot1 is an important enzyme in fatty acid metabolism; it catalyzes the reaction of fatty acyl-CoA to CoA-SH and free fatty acids. This role is crucial to cardiomyocyte mitochondria that use fatty acid for energy and ROS generation (Jezek and Hlavata 2005). Because Acot1 is an inhibitor of oxidative stress in cardiomyocytes, its inhibition by DOX may contribute to ROS-mediated lipid peroxidation and ferroptosis in cardiac cells (Liu et al. 2020; Xia et al. 2015). Moreover, in a recent study by Ou et al. (2024), in vitro and in vivo investigations confirmed the role of ferroptosis in DOX-induced cardiotoxicity. In the study, iron accumulation increased and the levels of ferroptosis markers GPX-4 and SLC7A11 were markedly depressed, followed by impairment in lipid metabolism. Lipid metabolism dysfunction has been shown to adversely affect cardiomyocyte mitochondria and may lead to increased generation of ROS and subsequent lipid peroxides that provoke ferroptotic signalling (Jezek and Hlavata 2005).

It is noteworthy that the effects of CP and DOX on FTH and FTL gene/protein expression in in vitro models are inconsistent. CP reduced the gene expression of FTH, FSP1 and FTL in ferroptosis-induced ototoxicity in the HEI-OC1 cell line and nephrotoxicity in HK-2 and KM mice (Song et al. 2024a, b), whereas DOX upregulated the gene expression of FTH and FTL in ferroptosis-induced cardiotoxicity in the H9c2 cell line. Although different CTs were involved, there is a need for further mechanistic studies to clarify the effects of CT on key gene-proteins involved in intracellular iron metabolism (Table 1).

**Table 1:** Ferroptosis and ferroptosis markers in chemotherapy-induced toxicity

Chemotherapy	Organ toxicity	Study model	Ferroptosis findings	Reference
Cisplatin	Ototoxicity	*In vitro* cell line		Jian et al. 2021
Cisplatin	Nephrotoxicity	mouse and *in vitro* HK-2 cells		Li et al. 2023
Cisplatin	Nephrotoxicity	mouse and *in vitro* HK-2 cells		Zhou et al. 2022
Cisplatin	Nephrotoxicity	Mouse		Ikeda et al. 2021
Cisplatin	Nephrotoxicity	rat and *in vitro* LLC-PK1 cells		Baliga et al. 1998
Cisplatin	Nephrotoxicity	*In vitro* HK-2 cells		Lin et al. 2024
Cisplatin	Nephrotoxicity	HK-2 cell line		Hu et al. 2022b
Cisplatin	Nephrotoxicity	mouse and HK-2 cells		Hu et al. 2020
Cisplatin	Ototoxicity	HEI-OC1 cell line		Song et al. 2024a
Cisplatin	Acute kidney injury	*In vivo* mouse and *in vitro* HK-2 cells		Song et al. 2024b
Cisplatin	Acute kidney injury	*In vivo* mouse and *in vitro* HK-2 cells		Sun et al. 2024
Doxorubicin	Cardiotoxicity	*In vitro* H9c2 cells		He et al. 2023
Doxorubicin	Cardiotoxicity	Rat		Ouyang et al. 2023
Doxorubicin	Cardiotoxicity	Mouse		Tadokoro et al. 2020
Doxorubicin	Cardiotoxicity	Mouse		Liu et al. 2020
Doxorubicin	Cardiotoxicity	Mouse and neonatal rat cardiomyocytes		Ou et al. 2024

## Non-ferroptotic mechanisms in chemotherapy-induced toxicity

### Oxidative stress

One of the key ways by which CT exerts deleterious effects is by promoting oxidative stress (Foufelle and Fromenty 2016). Although oxidative stress has been thoroughly investigated in relation to ferroptosis, a type of programmed cell death linked to iron-dependent lipid peroxidation, there are a number of non-ferroptotic ways in which oxidative stress mediates the toxicity of chemotherapeutic drugs (Zhao et al. 2022). ROS, which comprise non-radical molecules like hydrogen peroxide and free radicals like superoxide anion and hydroxyl radical, are extremely reactive (Gavanji et al. 2023). In physiological states, ROS are generated as metabolic byproducts of cells and play a role in multiple signalling cascades. On the contrary, chemotherapeutic drugs have the potential to markedly increase ROS levels, which might result in oxidative stress if ROS generation overwhelms the antioxidant defence of the cell (Bhattacharya 2015). An instance is the anthracycline class of CT, which includes DOX and is known to produce ROS via redox cycling (Akhigbe et al. 2023; Famurewa et al. 2023; Adeyemi et al. 2024). DOX undergoes one-electron reduction to form a semiquinone radical, which subsequently binds with oxygen to generate superoxide anion and other ROS. Lipids, proteins and DNA are among the biological components that these ROS do harm. Damage to DNA can lead to strand breakage, mutations and cross-linking, all of which can negatively impact the viability and function of cells. Furthermore, lipid peroxidation damages cellular membranes, resulting in loss of membrane integrity and cell death (Zhu et al. 2016). Mitochondria are central to cellular energy production but are also significant sources of ROS. CTs such as CP and PTX can induce mitochondrial dysfunction, contributing to oxidative stress and cytotoxicity. CP, for instance, can accumulate in the mitochondria and bind to mitochondrial DNA (mtDNA), disrupting the mitochondrial electron transport chain (ETC). This disruption leads to an increase in electron leakage and the formation of superoxide anion (Cocetta et al. 2019). Furthermore, mitochondrial dysfunction impairs ATP production, leading to energy deficits that affect cellular processes. As a result of ETC breakdown, mitochondrial membrane potential is lost. This can also cause pro-apoptotic substances such as cytochrome c to be released into the cytosol, which activates the intrinsic pathway of apoptosis. Caspases, which are proteolytic enzymes that cause cell death by cleaving particular substrates, are activated in this pathway (Chistiakov et al. 2018). Moreover, the endoplasmic reticulum (ER) plays a role in calcium homeostasis, lipid production and protein folding. By interfering with these processes, chemotherapeutic medications can cause ER stress and an accumulation of unfolded or misfolded proteins in the ER lumen that triggers the unfolded protein response (UPR), a signalling network that aims to restore ER homeostasis. Nonetheless, if the stress is intense or persistent, the UPR could trigger apoptotic pathways (Bonsignore et al. 2023). Bortezomib, a proteasome inhibitor used in the management of multiple myeloma, exemplifies this mechanism. Bortezomib blocks the proteasome, which stops misfolded proteins from degrading and building up, causing ER stress. The resulting oxidative stress can further exacerbate cellular damage, as the ER is a significant source of ROS production during protein folding (Auner and Cenci 2015). Cells possess a robust antioxidant buffer system that inhibits oxidative injury (Akhigbe and Hamed 2021a). Together with non-enzymatic antioxidants such as GSH, this system also contains enzymatic antioxidants, including superoxide dismutase (SOD), glutathione peroxidase and catalase (Akhigbe and Ajayi 2020). Anti-cancer drugs can disrupt these defenses, augmenting oxidative stress and toxicity (He et al. 2017). For instance, the depletion of GSH, a major cellular antioxidant that is essential for detoxifying ROS and maintaining redox balance within the cell (Akhigbe and Ajayi 2020; Famurewa et al. 2018), is a common consequence of treatment with CT drugs such as cisplatin and analgesic drugs such as acetaminophen (paracetamol). Cisplatin can form adducts with GSH, depleting its levels and impairing its protective functions. This depletion not only increases susceptibility to oxidative damage but also affects the activity of GSH-dependent enzymes, further compromising the cell’s ability to counteract ROS (Cheng et al. 2021). Oxidative stress can also trigger inflammatory responses, contributing to the side effect toxicity of CT drugs. ROS activates several signalling factors or pathways such as nuclear factor-kappa B (NF-κB) and mitogen-activated protein kinases (MAPKs), which regulate the transcription and release of inflammatory cytokines. The release of pro-inflammatory cytokines such as interleukins and tumour necrosis factor-alpha (TNF-α) exacerbates tissue injury and promotes cell death (Li et al. 2020a, b). MTX, a folate antagonist used in CT, induces inflammation and oxidative stress. MTX generates ROS that can activate NF-κB, leading to increased production of inflammatory cytokines. This inflammation can cause damage to normal tissues, contributing to the drug’s toxicity (Marin et al. 2022) (Fig. 4).

### Inflammation

Inflammation plays a crucial role in mediating the toxicity of CTs (Fig. 4). It is a complex biological response to harmful stimuli and can be both beneficial and detrimental (Cao et al. 2021). In the context of CT therapy, chronic inflammation induced by the drugs can lead to various adverse effects, including tissue damage, organ dysfunction and systemic toxicity (Vogel et al. 2020). Pro-inflammatory cytokines such as TNF-α, interleukin-1 beta (IL-1β) and interleukin-6 (IL-6) can be produced and released in response to CT (Famurewa et al. 2019). These cytokines initiate and amplify inflammatory responses that lead to various pathological conditions. For instance, DOX, an anthracycline antibiotic used in CT, is known to cause cardiotoxicity partly through the induction of inflammatory cytokines. DOX induces the expression of TNF-α and IL-1β in cardiac tissues, promoting an inflammatory environment that contributes to myocardial damage. The elevated cytokine levels activate downstream signalling pathways of drug-induced toxicity that exacerbate tissue injury and induce non-ferroptotic cell death (Musolino et al. 2017). The inflammatory response to CT drugs is mostly mediated by the immune system. These drugs can activate various immune cells, including macrophages, neutrophils and lymphocytes, which in turn release pro-inflammatory cytokines and chemokines. This activation can lead to acute and chronic inflammatory conditions (Zappavigna et al. 2020).

MTX activates macrophages resulting in the release of pro-inflammatory cytokines. The drug-induced activation of these immune cells can result in systemic inflammation, contributing to side effects such as mucositis, hepatotoxicity and nephrotoxicity. The inflammatory response triggered by immune cell activation could mediate the toxic effects of the CT drugs to lead to a non-ferroptotic form of cell death (De Freitas Saito et al. 2023). Multiple signalling pathways are involved in mediating the inflammatory response to anticancer CT drugs. Key among these are the nuclear factor-kappa B (NF-κB) and mitogen-activated protein kinase (MAPK) pathways. These pathways mediate CT-induced inflammation since they control the expression of genes that promote inflammation. The NF-κB pathway is a major regulator of inflammation and is often activated by anti-cancer CT drugs. The activation of NF-kB upregulates the expression of inflammatory cytokines and chemokines, contributing to anti-cancer CT drug-induced side effect toxicity. Similarly, the MAPK pathway, which includes ERK, JNK and p38 MAPK, is activated in response to stress signals and can mediate inflammatory responses to anti-cancer CT drugs (Chen et al. 2018).

Inflammation and oxidative stress are closely linked processes that can mutually reinforce each other. Anti-cancer CT drugs induce ROS release which result in oxidative stress that may activate various inflammatory signalling pathways, creating a feedback loop that amplifies both oxidative stress and inflammation (Yu et al. 2022a). CP, a platinum-based chemotherapeutic agent, is known to induce nephrotoxicity through mechanisms involving both oxidative stress and inflammation. CP generates ROS, which can activate the NF-κB and MAPK pathways, resulting in the generation of pro-inflammatory cytokines. These cytokines further exacerbate oxidative stress by recruiting immune cells that produce additional ROS, creating a vicious cycle of inflammation and oxidative damage. This interplay between oxidative stress and inflammation could underlie non-ferroptotic mechanisms of CP-induced toxicity (Sahu et al. 2014). The inflammatory response to anti-cancer CT drugs can vary, depending on the tissue(s) affected. Different tissues have distinct inflammatory environments and immune cell compositions, which can influence the nature and severity of drug-induced toxicity (Ramos-Casals et al. 2020). For example, the gastrointestinal tract is particularly susceptible to inflammation induced by chemotherapeutic drugs such as 5-fluorouracil (5-FU). 5-FU can cause mucositis, an inflammatory condition characterized by ulceration and pain in the mucosal lining of the digestive tract. The drug induces the release of pro-inflammatory cytokines and chemokines and the accumulation of immune cells and subsequent tissue damage. The localized inflammation in the gastrointestinal tract exemplifies how tissue-specific factors contribute to the side effect toxicity of CT drugs (Sim et al. 2023). In some cases, CT drugs can induce a systemic inflammatory response, leading to a condition known as systemic inflammatory response syndrome (SIRS). SIRS is characterized by widespread inflammation that can result in multiple organ failure, and is a severe complication of CT (Dolan et al. 2017). Taxanes, such as PTX, are known to cause SIRS. PTX can stimulate the release of high levels of pro-inflammatory cytokines, leading to systemic inflammation. The excessive inflammatory response can affect multiple organs, including the liver, kidneys and lungs, leading to widespread tissue damage and organ dysfunction. SIRS represents an extreme manifestation of drug-induced inflammation and highlights the potential outcomes of systemic side effect toxicity with cytotoxic anticancer therapies. However, the anti-inflammatory and/or protective roles of cytokines, including IL-4, IL-10, IL-19 and IL-22, have been shown in the existing literature, especially in the pathogenesis of cardiovascular diseases (Haybar et al. 2023; Algefare et al. 2024).

### Mitochondrial damage and apoptosis

One of the critical pathways through which CT drugs exert their side effect toxicity is by causing mitochondrial damage and inducing apoptosis, which is distinct from ferroptosis (Li et al. 2020a, b). Apoptosis, or programmed cell death, is a tightly regulated process that enables the body to remove damaged or unwanted cells in a controlled manner. Contrary to necrosis, which is a largely random cell death from acute cellular injury, apoptosis is a more orderly process that avoids eliciting an inflammatory response. Many CT drugs leverage apoptosis to kill cancer cells; however, they can also inadvertently trigger apoptosis in healthy cells, leading to side effect toxicity (Mishra et al. 2018). CT drugs can induce apoptosis through the intrinsic/mitochondrial and extrinsic/death receptor pathways (Pfeffer and Singh 2018; Hamed et al. 2024). The intrinsic pathway is initiated by intracellular signals, usually in response to oxidative stress, DNA damage or other forms of cellular stress. CT drugs like DOX and CP can induce significant DNA damage and oxidative stress, resulting in the activation of the intrinsic apoptotic pathway. This promotes the release of cytochrome c from the mitochondria into the cytosol, and procaspase-9, apoptotic protease activating factor-1 (Apaf-1) and cytochrome c create a complex that activates caspase-9. Activated caspase-9 then triggers a cascade of downstream caspases, such as caspase-3, which execute apoptosis by cleaving cellular proteins and dismantling the cell (Rezatabar et al. 2019).

The extrinsic pathway is activated by the interaction of extracellular death ligands with their corresponding death receptors on the cell surface (Akhigbe et al. 2021b). Anti-cancer agents like TRAIL (TNF-related apoptosis-inducing ligand) can engage this pathway. TRAIL binds to death receptors such as DR4 and DR5 to produce death-inducing signalling complex (DISC) that in turn activates caspase-8. Caspase-8 directly activates downstream effector caspases or cleaves Bid, a pro-apoptotic Bcl-2 family member, to tBid, to drive the intrinsic pathway (Jan 2019). However, mitochondria are the powerhouse of the cell, playing a crucial role in energy production, regulation of metabolic pathways and initiation of apoptosis. Many anti-cancer drugs target mitochondrial function, leading to mitochondrial damage, which contributes to their cytotoxic effects (Neuzil et al. 2014). Anti-cancer drugs such as CP can accumulate in the mitochondria and bind to mitochondrial DNA (mtDNA), causing cross-linking and mutations. This damage suppresses the mitochondrial electron transport chain (ETC), resulting in increased ROS production that further damages mtDNA, proteins and lipids, creating a vicious cycle of mitochondrial dysfunction and oxidative stress (Mapuskar et al. 2021). Drugs such as DOX can disrupt the mitochondrial membrane potential (Δψm). DOX interacts with cardiolipin, a phospholipid unique to the inner mitochondrial membrane, affecting the integrity of the membrane and the function of ETC complexes. Mitochondrial apoptosis is characterized by the loss of Δψm, which opens the mitochondrial permeability transition pore (mPTP) and releases pro-apoptotic substances such as cytochrome c and apoptosis-inducing factor (AIF) (Rocca et al. 2022). Mitochondrial damage results in impaired ATP production, leading to an energy deficit in the cell. ATP is essential for various cellular processes, including ion homeostasis and protein synthesis. The depletion of ATP can lead to cellular dysfunction and death, particularly in tissues with high energy demands, such as the heart and the brain. For example, anthracyclines such as DOX are notorious for causing cardiotoxicity through this mechanism (Seungyoon and Pekkurnaz 2018). Mitochondrial dysfunction often leads to the overproduction of ROS. Anti-cancer drugs such as PTX can exacerbate this effect by inhibiting microtubule dynamics, which are critical for mitochondrial transport and distribution within cells. Accumulated ROS can damage mitochondrial and nuclear DNA, proteins and lipids, further impairing cellular function and viability (Khan et al. 2022). Mitochondrial damage and apoptosis are closely interconnected. Mitochondrial dysfunction can lead to the release of apoptogenic factors, while the activation of apoptotic pathways can exacerbate mitochondrial damage (Belhadj Slimen et al. 2014).

The Bcl-2 protein family regulates apoptosis and mitochondrial integrity. This family comprises both pro-apoptotic (Bax, Bak) and anti-apoptotic (Bcl-2, Bcl-xL) proteins. Anti-cancer drugs can alter the balance between these proteins, tipping the scale towards apoptosis. For instance, drugs like venetoclax, a Bcl-2 inhibitor, promote the activation of Bax and Bak, mitochondrial outer membrane permeabilization (MOMP) and cytochrome c release (D'Orsi et al. 2017). The opening of the mPTP is a critical event in mitochondrial-mediated apoptosis. Drugs that cause oxidative stress or disrupt mitochondrial calcium homeostasis can trigger mPTP opening. For example, cisplatin induces mitochondrial calcium overload and oxidative stress, leading to mPTP opening and the release of pro-apoptotic factors. Once opened, the mPTP allows the passage of solutes across the mitochondrial membrane, leading to mitochondrial swelling, loss of Δψm and release of cytochrome c (Waseem and Wang 2023).

### Autophagy and other signalling alterations

Autophagy, a cellular degradation and recycling process, and other signalling alterations play critical roles in maintaining cellular homeostasis. When disrupted by anti-cancer drugs, these processes can lead to significant toxicity (Chen et al. 2021a). Some anti-cancer drugs can induce excessive autophagy, leading to autophagic cell death, also known as type II programmed cell death. For instance, drugs like temsirolimus, an mTOR inhibitor, can upregulate autophagy. While this can be beneficial in targeting cancer cells, excessive autophagy in normal cells can lead to the degradation of essential cellular components and cell death. The removal of crucial organelles and proteins can disrupt cellular functions, resulting in toxicity in healthy tissues (Cuomo et al. 2019). Conversely, some drugs inhibit autophagy, preventing cells from clearing damaged organelles and proteins, leading to cellular dysfunction and death. Chloroquine and hydroxychloroquine, for example, inhibit the fusion of autophagosomes with lysosomes. This inhibition can cause the accumulation of damaged cellular components, inducing stress and apoptosis in non-cancerous cells, thereby contributing to toxicity (Khandia et al. 2019).

Autophagy is controlled by several signalling pathways, including the PI3K/Akt/mTOR pathway, the AMPK pathway and the Beclin-1 complex. Anti-cancer drugs can disrupt these pathways, leading to altered autophagic activity. For example, the inhibition of the PI3K/Akt/mTOR pathway by drugs like rapamycin can lead to increased autophagy. While this might help in killing cancer cells, it can also result in unintended toxicity in normal cells. Apart from autophagy, several other cell signalling pathways are affected by anti-cancer drugs, contributing to their toxic effects. These include the NF-κB, mitogen-activated protein kinase (MAPK), p53 and Janus kinase/signal transducer and activator of transcription (JAK/STAT) signalling pathways. NF-κB is a critical regulator of immune response, inflammation and cell survival. Anti-cancer drugs can aberrantly activate or inhibit this pathway, leading to toxicity (Chen et al. 2021b). Some drugs, such as doxorubicin, activate the NF-κB pathway, leading to the transcription of pro-inflammatory cytokines and survival genes. Chronic activation of NF-κB can cause inflammation and contribute to drug resistance in cancer cells. In normal tissues, this can result in inflammatory damage and toxicity, as seen in doxorubicin-induced cardiotoxicity. Conversely, drugs that inhibit NF-κB signalling can impair cell survival mechanisms, making normal cells more susceptible to apoptosis. This can be particularly detrimental in tissues requiring high turnover or repair capacity, such as the gastrointestinal tract and the bone marrow (Mortezaee et al. 2019).

The MAPK pathway is involved in regulating cell growth, differentiation and apoptosis. CT anti-cancer drugs can disrupt MAPK signalling, leading to toxicity (Braicu et al. 2019). Drugs like CP can activate the MAPK pathway, leading to increased apoptosis. While this is beneficial in eliminating cancer cells, the activation of MAPK in normal cells can result in unwanted cell death and tissue damage. CP-induced nephrotoxicity is a prime example of MAPK activation leading to renal cell apoptosis and kidney damage (Achkar et al. 2018). On the other hand, inhibition of MAPK signalling by drugs such as MEK inhibitors can suppress cell proliferation and survival. In normal tissues, this inhibition can impair cellular repair and regeneration processes, leading to toxicity in rapidly dividing tissues such as the skin and the gastrointestinal mucosa (Kun et al. 2021). Also, the p53 tumour suppressor pathway is crucial for regulating the cell cycle and apoptosis in response to DNA damage. Anti-cancer drugs often target this pathway to induce cancer cell death, but they can also affect normal cells. Drugs like etoposide induce DNA damage and activate p53, leading to cell cycle arrest and apoptosis. While this is effective against cancer cells, normal cells with high turnover rates, such as hematopoietic stem cells, can also undergo apoptosis, leading to side effects like myelosuppression and immunosuppression (Karimian et al. 2016). Some drugs might inadvertently inhibit p53 signalling, reducing the cell’s ability to undergo apoptosis in response to DNA damage. This can lead to the accumulation of damaged cells, increasing the risk of secondary malignancies and other toxicities (Dobbelstein and Sorensen 2015). The JAK/STAT pathway is involved in cytokine signalling and immune regulation. Anti-cancer drugs can modulate this pathway, leading to various toxic effects. Cytokine therapies, such as interferons, activate the JAK/STAT pathway to boost the immune response against cancer. However, prolonged activation can result in systemic inflammation and autoimmune-like side effects, such as fatigue, myalgia and hepatotoxicity. Drugs that inhibit JAK/STAT signalling, like JAK inhibitors, can suppress immune function, increasing the risk of infections and impairing the body’s ability to respond to cellular stress and damage. This immunosuppressive effect can lead to increased susceptibility to infections and delayed wound healing (Bose et al. 2020).

### Pharmacological effects of ginger, 6-gingerol and zingerone: an overview

**Fig. 5:**
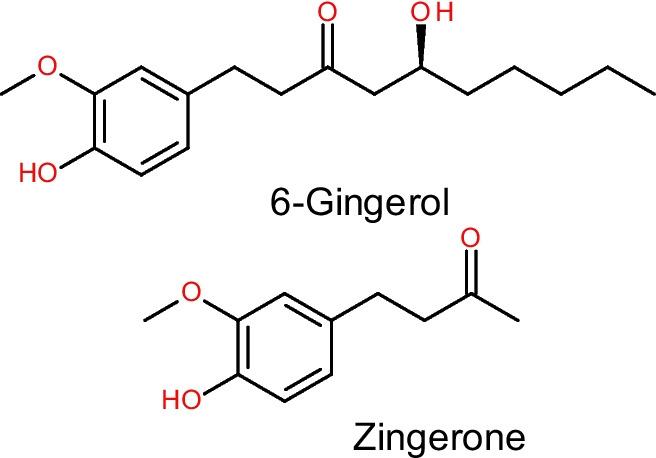
Chemical structures of 6-gingerol and zingerone

*Zingiber officinale* Roscoe (Ginger) (Zingiberaceae) is one of the most pungent spices which has been used extensively in Asia and Africa. From antiquity, it has been used to treat a variety of diseases (Ali et al. 2008). It is both cultivated widely as food and recognised for its healing properties in traditional medicine. Ginger has received enormous research attention, leading to investigating it for a wide range of pharmacological activities and isolating many bioactive compounds from it. The pharmacological activities of ginger include antioxidant, anti-inflammatory, antihypertensive, antibacterial, immunosuppressive, respiratory protective, neuroprotective and anticancer activities (Ali et al. 2008; Mao et al. 2019; Ali and Mamo 2023). Ginger contains many bioactive compounds, principally oleoresin and essential oil. The main components of oleoresin, the non-volatile pungent component, are identified as gingerols, which include 6-gingerol, 8-gingerol, 10-gingerol and 6-shogaol (the dehydrated product of 6-gingerol) and zingerone (Wang et al. 2009). 6-Gingerol (1-[4′-hydroxy-3′-methoxyphenyl]−5-hydroxy-3-decanone) and zingerone (4-(4-hydroxy-3-methylphenyl) butan-2-one) (Fig. 5) are important bioactive compounds isolated from the extracts of ginger and they show a wide range of pharmacological activities. This section focuses on an overview of the pharmacological effects of ginger and its bioactive compounds 6-gingerol (6G) and zingerone (ZG).

### Antioxidant activity

Eating foods high in antioxidants can protect against oxidative processes. Numerous naturally occurring antioxidants have been identified from a variety of plant materials, including seeds, fruits, vegetables, roots and leaves. Ginger and its phyto-constituents have been used to treat ROS-associated diseases. Ginger extract inhibits iron- and sodium nitroprusside (SNP)–induced lipid peroxidation in vitro (Akinyemi et al. 2013). Another study examined the protective effects of ginger extract against interleukin-1β (IL-1β)-induced oxidative stress and mitochondrial apoptosis in C28/I2 human chondrocytes. The cells were pre-treated with ginger extract for 24 h, followed by incubation with IL-1β for another 24 h to examine the protective effect of ginger extract on IL-1β-induced intracellular ROS production and lipid peroxidation. The results indicated that ginger extract pre-treatment significantly raised the gene expression of antioxidant enzymes (CAT, SOD-1, GPX-1, GPX-3 and GPX-4) and decreased the IL-1β-mediated rise of ROS, lipid peroxidation, the Bax/Bcl-2 ratio and caspase-3 activity (Hosseinzadeh et al. 2017). The effect of hydrogen peroxide-induced oxidative stress on HT1080 cell viability, ROS production, Akt activation and mitochondrial membrane potential was attenuated by ginger extract, resulting in decreased ROS production (at 200 and 400 µg/mL), increased mitochondrial membrane potential and decreased Akt activation (Romero et al. 2018). In addition to ginger extracts, 6G and ZG have also demonstrated antioxidant activities. Dugasani et al. (2010) compared the antioxidant activities of 6G, 8-gingerol, 10-gingerol and 6-shogaol in terms of scavenging of DPPH, superoxide and hydroxyl radicals. The four compounds demonstrated significant, concentration-dependent activities in all the models.

### Analgesic and anti-inflammatory activity

In an investigation into ginger’s anti-inflammatory properties, the herb inhibited T-lymphocyte Akt and NF-κB activation produced by anti-CD3 antibodies, as well as the TNFα-driven activation of epithelial NF-κB (Ueno et al. 2014). In an in vivo analgesic and anti-inflammatory study of 6G, its intraperitoneal administration at 25–50 mg/kg inhibited the acetic acid–induced writhing response and formalin-induced licking time in the late phase in male ICR and female Wistar rats. In addition, higher doses (50–100 mg/kg) of 6G inhibited carrageenan-induced paw oedema in the animals (Young et al. 2005). At 6 µM, 6G significantly inhibited N-formyl-methionyl-leucyl-phenylalanine-induced production of ROS in human polymorphonuclear neutrophils. It also inhibited the lipopolysaccharide (LPS)-induced production of inflammatory mediators (nitrite and prostaglandin E2) in RAW 264.7 cells in a dose-dependent manner (Dugasani et al. 2010).

### Anticancer activity

The literature has got a litany of data on the anticancer potential of ginger and its bioactive compounds. An investigation of the molecular mechanisms underlying the anticancer effects of an ethanolic extract of ginger in mice bearing solid Ehrlich carcinoma revealed that the extract’s activity was mediated by activation of adenosine monophosphate protein kinase (AMPK) and downregulation of the expression of cyclin D1 gene. An elevation of the p53 and suppression of the NF-κB levels in tumour tissue were also observed. In addition, the anticancer activity of DOX was potentiated by ginger extract (El-Ashmawy et al. 2018). A synergistic cytotoxicity of ginger extract with honey was observed in the colorectal cell line HT29. According to this study, the synergistic action of ginger and Gelam honey induced cell death in HT29 cells by upregulating caspase-9 and IκB genes, followed by a downregulation of KRAS, ERK, Akt, Bcl-xL and NFkB (p65) genes (Tahir et al. 2015). In vitro, in vivo and in silico studies have shown that 6G and ZG interfere with the carcinogenic processes. A study by Lee et al. (2008) showed that 6G inhibits the adhesion, invasion, motility and activities of MMP-2 and MMP-9 proteins in the human breast cancer cell line MDA-MB-231. Experimental data have indicated that 6G exerts its anticancer actions via important cell signalling mediators and pathways, which include Bax/Bcl-2, caspases‐3 and −9, extracellular signal-regulated protein kinases 1 and 2 (ERK1/2), nuclear factor erythroid 2-related factor-2 (Nrf2), p65/NF‐κB, p38/MAPK, p53, TNF‐α, ROS/NF‐κB/COX‐2 and SAPK/JNK. 6-Gingerol targets ferroptosis in lung cancer cells (A549 and CCD19-Lu cells; Balb/c nude mice) by downregulating ubiquitin-specific protease 14 (USP14), enhancing autophagy-dependent ferroptosis, increasing ROS and reducing the proliferation of cancer cells (Tsai et al. 2020). When phorbol 12-myristate 13-acetate (PMA)–treated human liver cancer cell lines (HepG2 and Hep3B) were incubated with 6G, the activity of matrix metalloproteinase-9 (MMP-9) was reduced, while an increase in the expression of tissue inhibitor metalloproteinase-1 (TIMP-1) protein was observed in both cell lines. These findings suggested that 6G exerts anti-invasive effect on liver cells through the regulation of MMP-9 (Weng et al. 2010). In the in vitro and in vivo investigation by Rastogi et al. (2015) of molecular mechanisms of action of 6G in human cervical cancer cells, at 50 µM, it potently inhibited the proliferation of the human papilloma virus (HPV)–positive cervical cancer cells (HeLa, CaSki and SiHa). In addition, 6G caused inhibition of the chymotrypsin activity of proteasomes, induction of reactivation of p53, increase in the levels of p21 and induction of ROS generation, leading to DNA damage and G2/M cell cycle arrest. It also altered the expression of p53-associated apoptotic markers (including cleaved caspase-3 and PARP), and enhanced the antiproliferative activity of CP. It increased the percentage of apoptotic cells in both early and late apoptotic phases in HeLa by 25.22%, in CaSki by 29.19% and in SiHa by 35.48%. In vivo, 6G treatment caused significant reduction of tumour volume, tumour weight, proteasome inhibition and p53 accumulation in HeLa xenograft tumour cells. 6G inhibited the growth of human cervical cancer cell line (HeLa) (IC_50_, 96.32 µM) through induction of cell cycle arrest at the G0/G1 phase by lowering the expression of cyclin (A, D1, E1) and upregulating the Bax/Bcl-2 ratio. It also caused activation of AMPK and inhibition of PI3K/Akt phosphorylation, with reduced expression of P70S6K and phosphorylation of mTOR. A combination of 6G with 5-FU and PTX led to much higher cellular inhibition compared to 6G alone (Zhang et al. 2017). HeLa cells treated with 6G showed depolarization of mitochondrial membrane potential, increased expression of caspase-3 and PARP, downregulation of NF-kB, Akt and Bcl2 gene expression, and upregulation of TNFα, Bax and cytochrome c protein expression. It also bound with the nuclear DNA and induced conformational changes to DNA in circular dichroism study. The study suggested that 6G could bind with DNA and mediate cell death by autophagy and caspase-3-induced apoptosis (Chakraborty et al. 2012). A study that investigated the action of 6G against human pancreatic cancer cell lines (HPAC expressing wildtype p53 and BxPC-3 expressing mutated p53) found that the compound inhibited cell growth via cell cycle arrest at the G1 phase in both cell lines. Further analysis by Western blot indicated that 6G reduced Cyclin A and Cyclin-dependent kinase expression. The expression of p53 was decreased in 6-gingerol-treated cells, suggesting that the induction of Cyclin-dependent kinase inhibitor (p21/cip1) was p53-independent (Park et al. 2006). 6-Gingerol pre-treatment in HepG2 cells has also partially protected against radiation-induced cell damage related to oxidative stress and apoptosis (Chung et al. 2015). In a colorectal cancer cell line (HCT116), 6G inhibited cancer cell growth by targeting leukotriene A_4_ activity. In vivo, it also suppressed tumour growth in nude mice (Jeong et al. 2009). Kang and co-workers evaluated the mechanism by which 6G induced cell death in non-small-cell lung cancer (NSCLC) cells. The results showed that 6G induced cell death by the mitochondrial-dependent, intrinsic apoptosis pathway. Iron metabolism was also found to play an important role in the antiproliferative potential of 6G through downregulation of EGFR/JAK2/STAT5b signalling or upregulation of p53 and downregulation of PD-L1 expression (Kang et al. 2023). ZG protected against radiation-induced DNA damage and apoptosis in lymphocytes in vitro. Pre-treatment of lymphocytes with ZG prior to exposure to gamma radiation was observed to significantly reduce the frequency of micronuclei, decrease genetic material and reduce percentage of apoptotic cells (Rao et al. 2011). In colon carcinogenesis induced by 1,2-dimethylhydrazine in Wistar rats, Ganaie et al. (2019) observed the protective effect of ZG via altering cytochrome P450 2E1 and serum marker enzyme carcinoembryonic antigen (CEA) activities, suppressing NF-kB-p65, COX-2, iNOS, PCNA and Ki-67 expression, and attenuating IL-6 and TNF-α levels. ZG also demonstrated anti-mitotic effect in human neuroblastoma cells (BE(2)-M17). ZG-treated neuroblastoma cells showed an increase in the number of mitotic cells, especially those in prometaphase, perhaps by decreasing the expression of cyclin D1 and inducing the cleavage of caspase-3 and PARP-1 (Choi et al. 2018). In PC-3 cells (prostate cancer model), ZG decreased cell viability in a concentration-dependent manner and induced apoptosis, as well as inhibited the PI3K/AKT/mTOR signalling pathway (Qian et al. 2021).

### Antimicrobial activity

Phyto-compounds are veritable sources of antibacterial agents (Berida et al. 2024). Ginger showed inhibitory effect against the growth of multi-drug-resistant *Pseudomonas aeruginosa* by altering its cellular physiology and inhibiting the biofilm formation (Chakotiya et al. 2017). Another study investigated, using a static biofilm assay, the potential of ginger extract to inhibit PA14 biofilm formation in *P. aeruginosa*. The results showed that ginger-treated culture had 39–56% reduction in biofilm development. In addition, treatment with ginger extract altered various phenotypes of PA14 (Kim and Park 2013). Crude extract and methanol fractions of ginger were found to inhibit various virulent properties of *Streptococcus mutans*. Biofilm development was reduced at critical growth phases, and glucan synthesis and adherence were also inhibited. In vivo, the extract and fractions also showed anti-cariogenic tendency in treated rats when compared to the untreated animals (Hasan et al. 2015). The effects of 6G on *Acinetobacter baumannii, Klebsiella pneumoniae*, *Pseudomonas aeruginosa* and *Staphylococcus aureus*, as well as on their biofilm formation and virulence properties, were investigated. For *S. aureus* and *P. aeruginosa*, 6G decreased efflux and damaged the cell membrane. It further decreased the biofilm formation and virulence factors production in *S. aureus* and *P. aeruginosa* at concentrations lower than the minimal inhibitory concentrations (MICs). Molecular dynamics simulation run for 100 ns also demonstrated a stable interaction of 6G with quorum sensing protein targets of *P. aeruginosa*. 6G’s ability to interfere with quorum sensing and virulence-regulating mechanisms in bacteria may be the cause of its anti-virulence characteristics (Elfaky et al. 2024). Zingerone has also demonstrated inhibitory effects against biofilm formation by *P. aeruginosa*. In addition, it improved the susceptibility of the bacterium to the quinolone antibiotic ciprofloxacin. Synergism was observed in the inhibition of *P. aeruginosa* biofilm formation when ZG was combined with ciprofloxacin (Kumar et al. 2013). The same researchers later investigated the anti-quorum sensing and anti-virulence potential of zingerone using *P. aeruginosa* PAO1. ZG decreased swimming, swarming and twitching phenotypes of *P. aeruginosa* PAO1, as well as reduced biofilm formation and production of virulence factors such as rhamnolipid, elastase, protease, pyocyanin and cell-free and cell-bound haemolysin (Kumar et al. 2015). Molecular docking analysis of ZG with quorum sensing receptors (TraR, LasR, RhlR and PqsR) was also carried out to gain insights into the mechanisms of its anti-virulence potential. *P. aeruginosa* was found to withstand high antibiotic concentrations in the presence of biofilm (Kumar et al. 2015). Therefore, overuse of antibiotics to treat biofilm-related infections has caused those antibiotics to lose their activity. Finding chemical compounds to inhibit biofilm formation is therefore a good research strategy which could also help reduce antibiotic resistance.

### Antihypertensive activity

The ability of ginger extract to inhibit angiotensin I-converting enzyme (ACE) and iron- and sodium nitroprusside (SNP)–induced lipid peroxidation was studied in vitro using rat isolated heart. The results showed that the extract inhibited ACE in a dose-dependent manner, with an IC_50_ value of 87.0 μg/mL. When the rat heart was incubated with Fe^2+^ and SNP, a marked rise in the level of MDA in the heart homogenates was observed. Upon addition of the ginger extract, a decrease in the MDA level in a dose-dependent manner was observed. This suggests that ginger exhibits its hypotensive effects through the inhibition of ACE activity and attenuation of lipid peroxidation (Akinyemi et al. 2013). Ginger extract blocks the voltage-dependent calcium channels to mediate lowering on blood pressure in different animal models (Ghayur and Gilani 2005). In spontaneously hypertensive rats, the hypotensive effect of ginger extract is suggested to be associated with vascular relaxation mechanisms via activation of cGMP-KATP channels, nitric oxide and prostacyclin release, transmembrane calcium channel or Ca^2+^ release from intracellular stores, and stimulation of muscarinic receptors (Razali et al. 2020).

### Cardioprotective activity

There is a significant death risk from myocardial ischemia/reperfusion injury because it severely damages the heart tissue. Myocardial ischemia/reperfusion damage is said to be significantly reduced by 6G. A study by Hosseini et al. (2024) shows that 6G has protective actions against lipopolysaccharide-induced cardiomyocyte injury in H9c2 cells through the suppression of ROS and pro-inflammatory cytokine production while enhancing GSH levels. Another significant study examined the underlying mechanisms by which 6G alleviates myocardial ischemia/reperfusion injury. 6G upregulated the expression of lncRNA H19 in hypoxia/reoxygenation-treated HL-1 cells. In addition, it elevated the expression of Bcl-2, while it reduced caspase 3 and caspase 9 levels. It was also observed that H19 both interacted with and lowered the cellular level of miR-143. The depletion of miR-143 by H19 caused an increase in the expression of its regulated gene (ATG7), which subsequently enhanced autophagy. According to the study’s findings, the H19/miR-143/ATG7 axis is essential to 6G’s ability to relieve myocardial ischemia/reperfusion injury (Lv et al. 2021). Network pharmacology and in vivo experimental data suggest that 6G can alleviate arsenic trioxide–induced liver injury and oxidative stress through the inhibition of pyroptosis and ROS-NLRP3 inflammatory signalling pathway (Wu et al. 2023). DOX, though used as an anticancer drug, has a major clinical limitation of being cardiotoxic. In a study by El-Bakly et al. (2012), it was shown that 6G pre-treatment notably ameliorated the elevated cardiac enzymes induced by DOX. The hepatoprotective effect of ZG in vivo was studied by inducing liver damage in rats using vancomycin. The hepatoprotective effect of ZG was attributed to its ability to regulate oxidative stress and inflammatory and apoptotic parameters (Kucukler et al. 2020).

### Neuroprotective activity

6G could also play a role in neurodegenerative diseases such as Alzheimer’s disease. The compound’s ability to alleviate neuroinflammatory effects in lipopolysaccharide (LPS)-induced disorder was studied using both in vitro and in vivo models. LPS-treated C6 astroglioma cells produced excessive pro-inflammatory cytokines (TNF-α and IL-6), as well as increased intercellular ROS, nitric oxide (NO) and inducible nitric oxide synthase (iNOS or NOS2). 6G inhibited all of them in a concentration-dependent manner. In vivo, the Morris water-maze (MWM) test was used to assess the rats’ spatial learning and memory after being exposed to LPS. A dose-dependent reduction in LPS-mediated impairment of MWM learning and memory was observed after exposure to 6G (Zhang et al. 2018a). Dose-dependent anticonvulsant activity of 6G was also observed in the pentylenetetrazole-induced hyperlocomotion assay in larval zebrafish, which occurred by decreasing the level of glutamic acid and glutamic acid/gamma-aminobutyric acid (GABA) ratio (Gawel et al. 2021). Asuku and co-workers investigated the protective effect of 6G-rich fraction on mercury chloride-induced neurotoxicity in vivo. The fraction inhibited mercury chloride–induced cognitive deficit by reducing brain inflammation and preventing oxidative stress in Wistar rats (Asuku et al. 2024). In addition, house dust mite–induced asthmatic oedema and inflammation of the bronchi and alveoli in a mouse model were prevented by the administration of 6G (Ajayi et al. 2022). Furthermore, Luo et al. (2021) reported that 6G demonstrated antiapoptotic and anti-inflammatory activities via TRPV1/FAF1 complex dissociation–induced autophagy during cerebral ischemia reperfusion injury. Similar findings were reported by Kongsui and Jittiwat (2023).

### Respiratory protective activity

Intraperitoneally injected LPS induced acute lung injury in Sprague–Dawley rats, which was characterized by elevated levels of inflammatory cells, alveolar haemorrhage and pulmonary interstitial oedema. 6G, given intraperitoneally, significantly reduced the lung wet/dry ratio and protein permeability index in the LPS-induced rats. It also suppressed oxidative and inflammatory damage by downregulating MDA, GSH, SOD, TNF-α, IL-6 and IL-1β levels in the lung tissue. In addition, 6G activated Nrf2/HO-1 signalling and suppressed LPS-induced NLRP3 inflammasome expression in lung tissues (Pan et al. 2023). Aqueous extract of fresh ginger was found to effectively reduce human respiratory syncytial virus (HRSV)–induced plaque formation in human HEp-2 and A549 respiratory tract cell lines in a dose-dependent manner (Chang et al. 2013). In vitro, 6G has an IC_50_ value of 2.25 ± 0.18 μM against the PR8 strain of the H1N1 influenza A virus. This activity was mediated by the inhibition of viral neuraminidase activity (Dutta et al. 2023). Studies have shown that, similar to 6G, ZG can attenuate LPS-induced production of proinflammatory cytokines in RAW 264.7 cells or LPS-induced acute lung injury in BALB/c mice (Xie et al. 2014).

### Anti-ulcerative colitis activity

6G shows potential to ameliorate ulcerative colitis through inhibition of ferroptosis, as evidenced through plasma metabolomics and network pharmacology analysis (Li et al. 2024). 6G suppressed the induction of ulcerative colitis in BALB/c mice exposed to dextran sulphate sodium (DSS) by promoting the activities of antioxidant enzymes and increasing glutathione level, and by decreasing H_2_O_2_ and MDA levels (Ajayi et al. 2015). Similarly, ZG was shown to attenuate ethanol-induced gastric ulcers in experimental rats. Gastric ulcers were induced in rats by oral ingestion of 5 mL/kg 96% ethanol. Oral administration of ZG at various doses significantly reduced both the mean number and length of gastric ulcers. The study concluded that the anti-ulcer effects of ZG may be mediated by its ability to scavenge free radicals in the rat stomach (Karampour et al. 2019).

#### Nephroprotective activity

Through its antioxidant and anti-inflammatory effects, 6G offers nephroprotective activity in streptozotocin-induced diabetes in rats. Administration of 6G to diabetic rats resulted in a considerable elevation in their kidneys’ levels of antioxidant enzymes and depressions in NF-kB protein expression, hyperlipidaemia and MDA levels (Almatroodi et al. 2021). Similarly, ZG too demonstrated reno-protective activity in LPS-induced acute kidney injury in vivo. The results indicate that ZG downregulated blood urea nitrogen, creatinine and TNF-α, IL-6 and IL-1β levels in a concentration-dependent manner (Song et al. 2016). ZG also protected against CP-induced nephrotoxicity in rat by suppressing oxidative stress and inflammation (Alibakhshi et al. 2018). Another study demonstrated that ZG could alleviate complications in alloxan-induced diabetic rats. Daily administration of ZG (50 and 100 mg/kg body weight) to diabetic rats for 21 days was found to suppress oxidative stress by enhancing the activities of GSH, SOD, CAT and GPx. It also suppressed NF-κB level and downregulated inflammatory cytokines (e.g. IL-1β, IL-2, IL-6) and TNF-α (Ahmad et al. 2018).

### Mechanisms of ginger’s mitigation of chemotherapy-induced side effect toxicity

Testicular dysfunction is one of the known adverse effects of CYP that may lead to infertility. Ahd et al. (2019) tested the efficacy of ginger extract against CYP-induced testicular toxicity in rats. The extract was administered at two doses (300 and 600 mg/kg). Treatment with ginger augmented germ cell count in the seminiferous tubules and testicular epithelium thickness. Besides, owing to its anti-oxidant activity, the extract buffered the oxidative imbalance induced by CYP, as evidenced by its effect on total anti-oxidant capacity. In addition, the extract at 600 mg/kg markedly enhanced testosterone level. Besides, treatment with the extract (fresh rhizomes of *Z. officinale*) at 300 mg/kg alleviated CYP-induced renal deficits in rats (Gabr et al. 2023). In the study, the extract reduced, at the gene expression level, the Bax/Bcl2 ratio and caspase-3, thus preventing CYP-induced apoptosis in the kidney tissue. It modulated renal microRNAs induced by CYP intoxication. Moreover, it upregulated the mRNA expression of Nrf2 and HO-1, as well as prevented renal fibrosis following CYP intoxication. Abdul-Hamid and Salah (2016) found that ginger (200 mg/kg) alleviated MTX-induced ileum toxicity in rats. In their study, ginger corrected the histological alterations induced by MTX. Furthermore, electron microscopy results revealed significant improvements in ileac cellular structures such as brush border length, nucleus, blood capillaries and mitochondria. The extract at 1 g/kg was found to counteract CP-induced reproductive toxicity in male rats (Amin and Hamza 2006). Treatment with the extract increased epididymal sperm count markedly. However, no significant effect was observed on oxidative stress markers, including CAT, SOD, GSH and MDA. Besides, histological examination revealed that animals pretreated with the extract displayed intact testicular morphology and spermatogenesis, with minor alterations in some tubules. In another study, Fekry et al. (2019) reported the protective effect of ginger against CP-induced testicular toxicity in male rats. Ginger treatment exhibited lower chromatin margination, prevented loss of germinal epithelium and reduced Leydig cell loss relative to the group of rats treated with CP only. Also, normal spermatogenic epithelium of seminiferous tubules was found in ginger-treated group. Moreover, in the ginger plus CP group, it was observed that there was a significant increase in serum testosterone level compared to CP only-treated group. Furthermore, ginger fresh juice extracted from ginger rhizomes was proved to counteract the toxic effects of CP in the rat testis (Famurewa et al. 2020a, b). Ginger juice was able to augment the levels of testosterone, LH and FSH in a significant manner following cisplatin administration. Moreover, the levels of inflammatory cytokines TNF-α, IL-1β and IL-6 were abrogated in testicular tissue following ginger juice administration. Ginger juice protected against CP-induced deficits in testicular tissue of rats, owing to its effect on the NO/iNOS/NF-κB signalling axis. However, the interleukin-10 family of cytokines have been implicated in the protection of delicate organs against toxicity. The pleiotropic cytokine IL-10 secreted by dendritic cells exerts an anti-inflammatory role, ameliorating CP-induced renal toxicity (Tadagavadi and Reeves 2010; Deng et al. 2001). An exogenous IL-10 mitigates ER stress in DOX-induced cardiac damage (Malik et al. 2022). In an in vivo model of CP-induced anorexia, ginger extract (100 and 500 mg/kg) enhanced total food and water intake, as well as body weights in rats (Kim et al. 2023). In addition, 5-HT level in nodose ganglion was found to be decreased following ginger extract treatment. This may be attributed to the effect of ginger extract on gene expression of 5-HT_3A_ and 5-HT_4_ receptors. These results were further confirmed by the protein expression results of both 5-HT_3A_ and 5-HT_4_ receptors at the right and left nodose ganglion. In another study, ginger extract protected against CP-induced cardiotoxicity in mice. Ginger extract administered at a dose of 500 mg/kg for 12 consecutive days exhibited less deposition of collagenous fibers in the cardiac tissue of mice compared to CP only-treated group. Also, the extract reduced the expression levels of p53 and TNF-α significantly. This was further confirmed by the morphometric analysis of the heart tissues. Ginger extract displayed euchromatic nucleus with some regular arrangement of myofibrils, in addition to intact, intercalated discs and Z lines (El-Hawwary and Omar 2019). One of the most known adverse effects of CP is nephrotoxicity. Ali et al. (2015) found that ginger extract administered at 120 mg/kg might protect against CP-induced kidney injury in rats. The extract induced a significant enhancement in the body weight of rats. In addition, it rescued renal tissues as evident by its effect on serum creatinine and blood urea nitrogen. Moreover, a marked decrease in the collagenous fibers was found in both the renal corpuscles and the tubules treated with ginger extract. The anti-apoptotic effect of the extract was confirmed by its downregulation of Bax protein expression. 6-Shogaol, an active constituent of ginger, exhibited protective activity against CP-induced renal injury in rats (Gwon et al. 2021). Treatment with 6-shogaol ameliorated CP-induced kidney injury as evidenced by reduced serum levels of creatinine and blood urea nitrogen. Also, NGAL and KIM-1 expression levels were found to be reduced with 6-shogaol treatment. Owing to its anti-oxidant property, 6-shogaol treatment alleviates renal oxidative damage induced by cisplatin as shown by decreased levels of MDA and 4-NHE, as well as enhanced GSH/GSSG ratio. Furthermore, 6-shogaol treatment revealed downregulated mRNA levels of iNOS, COX-2, 5-LOX and NOX-4. In a TUNEL assay of kidney tissues, a marked reduction in apoptosis level was found following 6-shogaol treatment relative to CP only-treated group. Besides, necroptosis proteins RIPK1, RIPK3 and p-MLKL were found to be downregulated with 6-shogaol treatment. A marked decrease in serum inflammatory markers was also seen following 6-shogaol treatment. One of the constituents of ginger extract, 10-dehydrogingerdione (10-DHGD), was found to alleviate CP-induced nephrotoxicity in rats (Elseweidy et al. 2016). CP treatment induced a marked increase in serum levels of creatinine, urea and total protein. This was opposed by 10-DHGD treatment, enhancing kidney function in rats. In addition, 10-DHGD showed a powerful anti-oxidant activity evident by decreased renal lipid peroxidation level and increased reduced glutathione level. Moreover, renal fibrosis markers such as fibroblast growth factor-23 and insulin-like growth factor I were found to be inhibited following 10-DHGD treatment. Regarding inflammatory response in renal tissues, 10-DHGD opposed the deleterious effect of cisplatin via downregulation of NK-κB, TNF-α and TGF-β. Extract of *Alpinia officinarum*, a rhizome from the ginger family, is traditionally used in Asia for its anti-inflammatory and antihyperlipidemic activities. CP use has been associated with hepatotoxicity, limiting its use in cancer patients. It was found that treatment with the *A. officinarum* extract prevented CP-induced toxicity by modulation of hepatotoxicity markers, oxidative stress, Bcl2 and p53 (Niazvand et al. 2023). In an animal model of oxaliplatin-induced peripheral neuropathy, *Z. officinale* rhizomes upregulated 5-HT_1A_ mRNA expression, confirming the inclusion of 5-HT_1A_, but not 5-HT_2A_, in the analgesic effect of *Z. officinale* (Lee et al. 2021). In another study, Kim et al. (2022) reported the protective effect of 6-shogaol against oxaliplatin-induced neuropathic pain in mice. Administration of 6-shogaol at 10 mg/kg, but not at 1 mg/kg, reduced mechanical and cold allodynia. This analgesic effect was found to be mediated via the 5-HT1A, 5-HT3 and GABA_B_ receptors (Table 2).

**Table 2:** Mechanistic actions of ginger extract, 6G and ZG on CT-induced toxicity in different models

Drug	Model	Mechanisms of action	Effect	Dossage	References
Ginger (Zingiber officinale)	Japanese Quails	Ginger buffers oxidative and metabolic imbalance by causing reduction in plasma total cholesterol, lipid peroxidation and improving antioxidant defence	Augmentation of germ cells count in seminiferous tubules and thickness of testicular epithelium	10, and 15 g/kg feed, 6 weeks (wks), oral	Ahd et al. 2019
	Rat	Increase in MDA and decrease in total antioxidant status (TAC)	Prevents cadmium-induced renal toxicity	100 and 200 mg/kg, 4 wks, oral	Gabr et al. 2019
	Rat	Increased activities of antioxidant enzymes malondialdehyde (MDA), with associated decrease in nitric oxide, NF-ĸB-dependent inflammation	It prevents cisplatin-induced testicular toxicity and injury and increase in serum testosterone level	100–500 mg/kg, 6 wks, oral; 5 days, oral	Essawy et al. 2018; Famurewa et al. 2020a, b
	Rat	Ginger abrogates the levels of inflammatory cytokines such as TNF-α, IL-1β and IL-6 in testicular tissue	Ginger induces significant enhancement in body weight of rats	120 mg/kg, 4 wks, oral; 3 days, ip	Ali et al. 2015; Gwon et al. 2021
	Mouse	By decreasing cardiac malondialdehyde, tumour necrosis factor-a and serum activity of creatine kinase and lactate dehydrogenase	It enhances cytoxocity and cardiotoxicty in hepatocellular carcinoma	60 KDa, 3 wks, ip	Abo Mansour et al. 2021
	Mouse	By inducing ginsenoside Rg3 (Rg3)	It decreases cold and mechanical allodynia by inhibiting spinal noradrenergic system in mice	100–300 mg/kg, 5 days, oral	Park et al. 2023
6-Gingerol	Mouse	By decreasing mitogen-activated protein kinase p38 (p38) phosphorylation	It prevents cardiac cellular damage resulting from cardiac hypertrophy, fibrosis, inflammation and dysfunction in TAC mice	20 mg/kg, 4 wks, oral	Ma et al. 2021
	Cell line	Modulation of caspase 3/γH2AX and Cdk-6 cyclin	It reduced doxorubicin-induced weight loss and hepatotoxicity in tumour-bearing animals	10 mg/kg, 4 wks, oral	Baptista Moreno Martin et al. 2020
	Wistar rat	6GBy modulated cardiac sRAGE/NF-κB/caspase 3 signalling in doxorubicin-treated Wistar rats and attenuated cardiac injury	6G attenuated doxorubicin-induced cardiotoxicity	10 mg/kg, 2 wks, oral	El-Bakly et al. 2012
	H9c2 cardiomyocytes	6G activates PPARα signalling in doxorubicin therapy, thus upregulates PPARα/PGC-1α/Sirt3-related mRNA and protein expression	6G attenuated doxorubicin-induced mitochondrial dysfunction and toxicity in H9c2 cells		Wen et al. 2019
	Hepatocellular carcinoma cells	By decreasing inflammatory cytokines, including TNF-α and IL-6, and also increase intercellular reactive oxygen species, nitric oxide and nitric oxide synthase	It ameliorated renal injury and preserved the histological structure of the kidneys	20 mM	Hu and Zhao 2021
Zingerone (ZG)	Wistar rat	ZG abates oxidative damage and DNA damage caused by cisplatin therapy, thus improves lipid profile and hepatic injury markers	ZG protects cisplatin-induced hepatotoxicity DNA damage	50 mg/kg, 2 wks, oral	Mir et al. 2018
	Sprague–Dawley rat	ZG abated MDA generation and oxidative stress, suppressed nitric oxide and 8OHdG generation and attenuated caspase 3-mediated apoptosis	ZG protects against cisplatin-induced hepatotoxicity	25 mg/kg and 50 mg/kg, oral	Kandemir and Mahamadu 2021
	Swiss Albino Mouse	By decreasing MDA, NO, COX-2, PGE_2_, TNF-α and IL-1β	ZG exhibits potential nephroprotective effect toward ADM-mediated nephrotoxicity with improved creatinine, urea, LDH. ZG preserves renal histoarchitecture and renal function	25 mg/kg, oral, 3 wks	Elshopakey et al. 2022
	Rat	By decreasing MDA, NO, 8’ OHDG, AND pro-inflammatory cytokines (comprising tumor necrosis factor-α, interleukin-1β, interleukin-6 and nuclear factor kappa B) as well as myeloperoxidase activity, and hence ZG suppresses inflammation induced by ADM. Zingerone reversed the changes in levels and/or activities of inflammatory and apoptotic parameters such as NF-κB, TNF-α, IL-1β, iNOS, COX-2), p53, caspase-3, caspase-8, cytochrome c, Bax, and Bcl-2 in the VCM-induced hepatotoxicity	ZG downregulated oxidative stress, inflammation, and apoptosis in vancomycin-induced hepatotoxicity	25 and 50 mg/kg, oral, 1 wk	Kucukler et al. 2020
	Rat	Suppression of the levels of MDA, TNF-α, and elevation of the renal activities of antioxidant enzymes	ZG reduces oxidative stress and inflammation against cisplatin-induced nephrotoxicity	10, 20 and 50 mg/kg, oral, 1 wk	Alibakhshi et al. 2018

Red ginger extract administered at 100 and 300 mg/kg in rats exhibited significant protection against oxaliplatin-induced neuropathy (Park et al. 2023). Pretreatment with red ginger extract markedly decreased cold and mechanical allodynia starting from day 3 and continuing to day 5 post-oxaliplatin exposure. Moreover, the analgesic effect of red ginger extract may be related to its effect on spinal noradrenergic system. Treatment of rats with the extract reduced the mRNA expression levels of α1- and α2-adrenergic receptors, as well as serum and spinal noradrenaline levels. In a tumour-bearing mouse model, ginger extract nanoparticles ameliorated cardiotoxicity induced by DOX (Abo Mansour et al. 2021). In an MTT assay, ginger extract nanoparticles enhanced the cytotoxic anticancer activity of DOX. Furthermore, liver index and serum transaminase activities of ginger extract–treated rats were found restored comparable to normal rats. Serum cardiotoxicity markers CK-MB and LDH were reduced following ginger extract nanoparticle treatment. Moreover, ginger extract loaded into chitosan nanoparticles showed normal levels of GSH and TNF-α, as well as gene expression of GPx, CAT and MDR1. Normal levels of liver content of VEGF and Bcl2 were noticed in ginger nanoparticle–treated group. An aqueous ethanol extract of the rhizome of *Z. officinale* (200 and 400 mg/kg) prevented DOX-induced nephrotoxicity in female rats (Ajith et al. 2008). In a dose-dependent manner, *Z. officinale* extract reduced serum levels of urea and creatinine, compared to DOX only–treated group. In addition, renal activities of CAT, SOD and GSH were found to be enhanced following ginger extract treatment. Also, the treatment inhibited lipid peroxidation level markedly, relative to DOX only–treated rats.

### Mechanisms of 6-gingerol’s mitigation of chemotherapy-induced side effect toxicity

6-Gingerol (6G), a key bioactive component of ginger, has received great interest for its possible health advantages, which include anti-inflammatory, antioxidant and anti-cancer effects. 6G’s natural properties may offer a means to alleviate these adverse effects, enhancing patient quality of life and potentially improving treatment outcomes (Srinivasan et al*.* 2019). Chemotherapeutic agents such as DOX, CP and CYP are known to generate ROS. While ROS are useful in killing cancer cells, they also harm normal cells, causing oxidative stress that leads to lipid peroxidation, protein damage and DNA mutations. These effects manifest as cardiotoxicity, nephrotoxicity, hepatotoxicity and neurotoxicity (Sahoo et al. 2022). 6G effectively scavenges free radicals, including superoxide anions, hydroxyl radicals and peroxyl radicals. By neutralizing these ROS, 6G reduces oxidative stress and prevents cellular damage. Research has shown that 6G can significantly decrease levels of malondialdehyde (MDA), a marker of lipid peroxidation, thereby preserving cellular integrity (Tahoun et al. 2021). 6G enhances the body’s endogenous antioxidant defenses by upregulating key enzymes such as SOD, GPx and CAT. These enzymes neutralize ROS and confer cellular protection against oxidative damage. SOD converts the superoxide radical into hydrogen peroxide, which is then broken down by CAT and GPx into water and oxygen, reducing oxidative stress (Akhigbe et al. 2021c; Ballester et al. 2023). The nuclear factor erythroid 2–related factor 2 (Nrf2) pathway is a major regulator of the cellular antioxidant response. Under oxidative stress, Nrf2 translocates to the nucleus, activating the expression of various antioxidant and cytoprotective genes. 6G activates the Nrf2 pathway, enhancing the cellular capacity to detoxify and eliminate ROS, thus maintaining redox homeostasis and protecting tissues from oxidative damage induced by CT (Zhao et al. 2017). DOX, a widely used chemotherapeutic agent, is known for its cardiotoxic effects, mediated primarily through oxidative stress. 6G’s antioxidative property can mitigate DOX-induced cardiotoxicity. Studies in animal models have shown that 6G reduces markers of cardiac injury such as creatine kinase-MB (CK-MB) and lactate dehydrogenase (LDH) by decreasing oxidative stress in cardiac tissues. Additionally, 6G improves the histopathological features of the heart, indicating reduced tissue damage (Abushouk et al. 2017).

Cisplatin (CP), another common CT, induces severe nephrotoxicity through the generation of ROS and subsequent oxidative damage in renal tissues. 6G’s antioxidative mechanisms can alleviate CP-induced nephrotoxicity. Research has demonstrated that 6G reduces levels of blood urea nitrogen (BUN) and serum creatinine, markers of kidney injury, by attenuating oxidative stress in renal tissues. 6G also preserves the histological structure of the kidneys, indicating its protective effects against CP-induced renal damage (Alibakhshi et al. 2018). 6G exerts strong anti-inflammatory effects by inhibiting key pro-inflammatory pathways such as the NF-κB pathway. NF-κB is a critical regulator of inflammation and is often upregulated in response to chemotherapy. By inhibiting NF-κB, gingerol reduces the production of inflammatory cytokines such as TNF-α, IL-1β and IL-6, which are implicated in CT-induced inflammation and tissue damage. This anti-inflammatory action not only protects normal tissues but can also reduce symptoms such as mucositis and enteritis, common in patients undergoing CT (Yücel et al. 2022).

While apoptosis is a desired outcome in cancer cells, its induction in healthy cells contributes to the side effects of CT. 6G has been shown to modulate apoptosis pathways selectively. In cancer cells, 6G induces apoptosis by activating pro-apoptotic proteins and inhibiting anti-apoptotic proteins. However, in normal cells, 6G appears to exert a protective effect by enhancing cell survival pathways and inhibiting apoptosis. This dual action helps to minimize the damage to healthy tissues during CT (Zadorozhna and Mangieri 2021). 6G can also influence the metabolism and pharmacokinetics of chemotherapeutic drugs. It has been found to modulate the activity of cytochrome P450 enzymes, which are involved in drug metabolism. By altering the activity of these enzymes, 6G can potentially reduce the formation of toxic metabolites associated with chemotherapy. This modulation can help in lowering the overall toxicity of the drugs without compromising their efficacy against cancer cells (Mukkavilli et al. 2014).

Gastrointestinal toxicity is a common side effect of several chemotherapeutic agents, often leading to severe nausea, vomiting and diarrhea. 6G has been shown to have gastroprotective effects, largely attributed to its anti-inflammatory and antioxidant properties. It protects the gastric mucosa and reduces the secretion of gastric acid, thereby preventing ulceration and other forms of gastrointestinal damage. Furthermore, 6G’s ability to accelerate gastric emptying and improve gastrointestinal motility can alleviate nausea and vomiting, thus significantly improving patient comfort and adherence to CT regimens (Balogun et al. 2019). Neurotoxicity is another significant adverse effect of certain chemotherapeutic agents, such as platinum-based drugs. 6G’s neuroprotective properties are thought to be mediated through its antioxidant and anti-inflammatory actions. By reducing oxidative stress and inflammation in neural tissues, 6G can protect against CT-induced peripheral neuropathy, a common and often debilitating side effect. Studies have demonstrated that 6G can enhance the expression of neurotrophic factors like brain-derived neurotrophic factor (BDNF), thus promoting neuronal survival and regeneration (Cocetta et al. 2023) (Table 2).

### Mechanisms of zingerone’s mitigation of chemotherapy-induced side effect toxicity

Zingerone (ZG), a bioactive compound found in ginger, has garnered significant attention for its potential therapeutic applications. One area of growing interest is its ability to mitigate the side effect toxicity of anti-cancer drugs. CT, while effective in combating cancer, often causes severe side effects due to the induction of oxidative stress and inflammation in normal tissues. ZG, via its antioxidant and anti-inflammatory properties, offers a promising approach to reducing these toxic side effects (Shamsabadi et al. 2023). CTs such as DOX, CP and CYP are widely used to treat various cancers. However, these drugs are associated with significant toxicities. DOX induces oxidative stress and damages cardiac tissues, leading to cardiomyopathy and heart failure. CP causes nephrotoxicity by generating ROS that damage renal cells, leading to kidney dysfunction. CYP induces hepatotoxicity and hemorrhagic cystitis through oxidative stress and inflammatory mechanisms. The toxicities associated with these drugs often limit their dosage and effectiveness, underscoring the need for adjunctive therapies that can mitigate these adverse effects (Famurewa et al. 2021; Gadisa et al. 2020).

The antioxidative property of ZG is central to its ability to mitigate CT-induced toxicity. ZG abates oxidative damage and inhibits cellular damage by combating ROS (Mir et al. 2018). ZG can directly neutralize ROS such as superoxide anions, hydroxyl radicals and peroxyl radicals. It increases the activities of endogenous antioxidant enzymes such as SOD, GPx and CAT. These enzymes play a critical role in detoxifying ROS, thus protecting cells from oxidative damage (Srinivasan 2014). ZG activates the Nrf2 pathway, a key transcription factor in cellular antioxidant response, to promote its translocation to the nucleus and enhance the expression of antioxidant response element (ARE)–driven genes, which encode for various antioxidant and cytoprotective proteins. This activation enhances the cellular capacity to combat oxidative stress (Wu et al. 2022). ZG also possesses potent anti-inflammatory properties. ZG mitigates inflammation and its associated damage via the inhibition of NF-κB activation, thereby reducing the expression of pro-inflammatory cytokines such as TNF-α, IL-1β and IL-6 (Bashir et al. 2021). More so, ZG suppresses the activities of enzymes like cyclooxygenase-2 (COX-2) and inducible nitric oxide synthase (iNOS), thus inhibiting the production of inflammatory mediators, e.g. prostaglandins and nitric oxide (Mehrzadi et al. 2021). DOX-induced cardiotoxicity is a major concern in cancer therapy. ZG reduces the levels of cardiac injury markers such as creatine kinase-MB (CK-MB) and lactate dehydrogenase (LDH) by attenuating oxidative stress in cardiac tissues (Al-Thubiani 2021). Furthermore, by upregulating the activities of antioxidant enzymes and reducing lipid peroxidation, ZG helps preserve cardiac function and structure. Histopathological studies in animal models revealed that ZG treatment attenuated cisplatin-induced myocardial damage and improved cardiac tissue integrity through the attenuation of oxidative stress and inflammatory response (Soliman et al. 2018). Elshopakey et al. (2022) revealed that ZG preserves renal histoarchitecture and renal function in adriamycin-exposed mice by suppressing pro-inflammatory cytokines. Additionally, ZG protected against CYP-induced hepatotoxicity (Mir et al. 2018) and vancomycin-induced hepatotoxicity (Kucukler et al. 2020) by mitigating oxidative stress and enhancing the activities of hepatic antioxidant enzymes.

## Research gaps and future perspectives

Ferroptosis is a regulated cell death characterized by iron dyshomeostasis and lipid peroxidation and contributes to the development of CT-induced organ toxicity. While this literature review explored the effects of ginger, 6G and ZG on ferroptosis in the context of CT-induced organ toxicity, research in that area is still emerging and should be strengthened. For example, whether ginger, 6G or ZG could modulate iron overload through transferrin receptor-1/ferritin/ferritinophagy axis remains unclear, and thus should be explored in greater details. Also, a systematic investigation of the possible effects of the natural agents on antiferroptotic SLC7A11 and SLC3A2 proteins and/or genes should be conducted. The crucial roles of microRNA, Wnt/β-catenin and lncRNAs signalling are emerging in CT-induced toxicity, and they are potential therapeutic targets in this regard, and hence an avenue for future research. Besides CP and DOX, research should explore the protective effects of ginger, 6G and ZG against side effect toxicities induced by other CT drugs. The potential for ginger, 6G and ZG to elicit chemotherapeutic efficacy should also be explored, for their ability to be used in combination or adjuvant anticancer therapy.

## Conclusions

The current review reveals the involvement of ferroptosis and non-ferroptotic oxidative stress, NF-κB-mediated inflammation, autophagy and apoptosis in the pathogenesis of CT-induced organ toxicity. It also shows, through the published studies, that ERK/JNK/p38MAPK and PI3K/Akt/mTOR signalling are allied pathways provoking CT-induced organ toxicity and damage. Antioxidant, anti-inflammatory and antiapoptotic processes are the chief mechanisms through which ginger, 6G and ZG could combat CT-induced organ toxicity. In vivo and in vitro models demonstrate that the side effect toxicities by CP and DOX are the most explored with respect to the therapeutic effects of ginger, 6G and ZG against such toxicities. Undoubtedly, more studies are required to evaluate the side effect toxicity mechanisms of other CT drugs, and the potential protection against them by natural products ginger, 6G and ZG, before clinical trials can be undertaken.

## Data Availability

All source data for this work (or generated in this study) are available upon reasonable request.

## Abbreviations

ACE: Angiotensin I-converting enzyme
ACSL4: Acyl‑CoA synthetase long‑chain family member 4
AIF: Apoptosis-inducing factor
Akt: Protein kinase B
ALOX: Arachidonate lipoxygenase
Apaf-1: Apoptotic protease activating factor-1
Bax: Bcl-2 associated X
Bcl-2: B cell lymphoma 2
BDNF: Brain-derived neurotrophic factor
BH4: Tetrahydrobiopterin
CoQ10: Coenzyme Q10
COX: Cyclooxygenases
CT: Chemotherapy
CYP: Cyclophosphamide
DHFR: Dihydrofolate reductase
ERK: Extracellular signal-regulated kinase
FSP1: Ferroptosis suppressor protein-1
FTH-1: Ferritin heavy chain-1
FTL-1: Ferritin light chain-1
GABA: Gamma-aminobutyric acid
GPX-4: Glutathione peroxidase-4
GSK: Glycogen synthase kinase
HO: Heme oxygenase
IL: Interleukins
iNOS: Inducible nitric oxide synthase
IκB: Kinase IKK
JNK: Jun N-terminal kinase
Keap1: Kelch-like ECH-associatedprotein 1
LncRNAs: LONG non-coding RNA
LOX: Lipoxygenase
MAPK: Mitogen-activated protein kinase
mPTP: Mitochondrial permeability transition pore
mTOR: Mammalian target of rapamycin
NCOA4: Nuclear receptor coactivator 4
NF-κB: Nuclear factor-kappa B
NLRP: Nod-like receptor
NOX: NADPH oxidase
Nrf2: Nuclear factor-erythroid factor 2
PI3K: Phosphatidylinositol-3 kinase
PLOOH: Phospholipid hydroxylperoxide
PPAR-α: Peroxisome proliferator-activated receptor-α
PUFA-CoA: Polyunsaturated fatty acid coenzyme-A
PUFAs: Polyunsaturated fatty acids
ROS/RNS: Reactive oxygen/reactive nitrogen species
SIRS: Systemic inflammatory response syndrome
SIRT1: Silent information regulator T1
SLC7A11: Light-chain subunit solute carrier family 7 member 11
SNP: Sodium nitroprusside
STAT: Signal transducers and activators of transcription
System Xc^−^: Glutamate-cysteine transporter system
TFR: Transferrin receptor
TLR-4: Toll-like receptor-4
TLR-4: Toll-like receptor-4
TNFα: Tumour necrosis factor-alpha
TRAIL: TNF-Related apoptosis-inducing ligand
UPR: Unfolded protein response
USP-14: Ubiquitin-specific protease-14
Wnt: Wingless-related integration site
Δψm: Mitochondrial membrane potential

## References

[CR1] Abdul-Hamid M, Salah M (2016) Intervention of ginger or propolis ameliorates methotrexate-induced ileum toxicity. Toxicol Ind Health 32(2):313–322 24097362 10.1177/0748233713500833

[CR2] Abo Mansour HE, El-Batsh MM, Badawy NS, Mehanna ET, Mesbah NM, Abo-Elmatty DM (2021) Ginger extract loaded into chitosan nanoparticles enhances cytotoxicity and reduces cardiotoxicity of doxorubicin in hepatocellular carcinoma in mice. Nutr Cancer 73(11–12):2347–2362 32972241 10.1080/01635581.2020.1823436

[CR3] Abushouk AI, Ismail A, Salem AM, Afifi AM, Abdel-Daim MM (2017) Cardioprotective mechanisms of phytochemicals against doxorubicin-induced cardiotoxicity. Biomed Pharmacother 90:935–946 28460429 10.1016/j.biopha.2017.04.033

[CR4] Achkar IW, Abdulrahman N, Al-Sulaiti H, Joseph JM, Uddin S, Mraiche F (2018) Cisplatin-based therapy: the role of the mitogen activated protein kinase signaling pathway. J Transl Med 16:1–2 29642900 10.1186/s12967-018-1471-1 PMC5896132

[CR5] Adeyemi DH, Obembe OO, Hamed MA, Akhigbe RE (2024) Sodium acetate ameliorates doxorubicin-induced cardiac injury via upregulation of Nrf2/HO-1 signaling and downregulation of NFkB-mediated apoptotic signaling in Wistar rats. Naunyn-Schmiedeberg’s Arch Pharmacol 397(1):423–435 37458777 10.1007/s00210-023-02620-4

[CR6] Ahd K, Dhibi S, Akermi S, Bouzenna H, Samout N, Elfeki A, Hfaiedh N (2019) Protective effect of ginger (Zingiber officinale) against PCB-induced acute hepatotoxicity in male rats. RSC Adv 9(50):29120–29130 35528415 10.1039/c9ra03136g PMC9071811

[CR7] Ahmad B, Rehman MU, Amin I, Rahman Mir M, Ahmad SB, Farooq A, Muzamil S, Hussain I, Masoodi M, Fatima B (2018) Zingerone (4-(4-hydroxy-3-methylphenyl) butan-2-one) protects against alloxan-induced diabetes via alleviation of oxidative stress and inflammation: probable role of NF-kB activation. Saudi Pharm J 26:1137–1145 30532634 10.1016/j.jsps.2018.07.001 PMC6260481

[CR8] Aisner J (2007) Overview of the changing paradigm in cancer treatment: oral chemotherapy. Am J Health Syst Pharm 64:S4-7 17468157 10.2146/ajhp070035

[CR9] Ajayi BO, Adedara IA, Farombi EO (2015) Pharmacological activity of 6-gingerol in dextran sulphate sodium-induced ulcerative colitis in BALB/c mice. Phytother Res 29:566–572 25631463 10.1002/ptr.5286

[CR10] Ajayi BO, Olajide TA, Olayinka ET (2022) 6-gingerol attenuates pulmonary inflammation and oxidative stress in mice model of house dust mite-induced asthma. Adv Redox Res 5:100036

[CR11] Ajith TA, Aswathy MS, Hema U (2008) Protective effect of *Zingiber officinale* roscoe against anticancer drug doxorubicin-induced acute nephrotoxicity. Food Chem Toxicol 46(9):3178–3181 18680783 10.1016/j.fct.2008.07.004

[CR12] Akhigbe R, Ajayi A (2020) Testicular toxicity following chronic codeine administration is via oxidative DNA damage and up-regulation of NO/TNF-α and caspase 3 activities. PLoS ONE 15(3):e0224052 32168344 10.1371/journal.pone.0224052 PMC7069647

[CR13] Akhigbe RE, Hamed MA (2021a) Co-administration of HAART and antikoch triggers cardiometabolic dysfunction through an oxidative stress-mediated pathway. Lipids Health Dis 20:1–5 34225751 10.1186/s12944-021-01493-x PMC8259328

[CR14] Akhigbe RE, Ajayi LO, Ajayi AF (2021b) Codeine exerts cardiorenal injury via upregulation of adenine deaminase/xanthine oxidase and caspase 3 signaling. Life Sci 273:118717 33159958 10.1016/j.lfs.2020.118717

[CR15] Akhigbe RE, Hamed MA, Aremu AO (2021c) HAART exacerbates testicular damage and impaired spermatogenesis in anti-Koch-treated rats via dysregulation of lactate transport and glutathione content. Reprod Toxicol 103:96–107 34118364 10.1016/j.reprotox.2021.06.007

[CR16] Akhigbe R, Adeyemi D, Hamed M, Akhigbe T (2023) P-008 Acetate ameliorates doxorubicin-induced testicular toxicity by modulating Nrf2/NFkB pathway and apoptotic signaling. Hum Reprod 38(Supplement_1):093–378

[CR17] Akinyemi AJ, Ademiluyi AO, Oboh G (2013) Aqueous extracts of two varieties of ginger (*Zingiber officinale*) inhibit angiotensin I-Converting enzyme, iron(II), and sodium nitroprusside-induced lipid peroxidation in the rat heart in vitro. J Med Food 16(7):641–646 23875904 10.1089/jmf.2012.0022

[CR18] Al-Amir HJ, Janabi AM, Kadhim SF, Wasfi RM (2023) Isosorbide dinitrate improves doxorubicin-induced cardiotoxicity via diminishing pro-inflammatory mediators, oxidative stress, and apoptosis. J Popul Ther Clin Pharmacol 30(5):295–306

[CR19] Algefare AI, Alfwuaires M, Famurewa AC, Elsawy H, Sedky A (2024) Geraniol prevents CCl_4_-induced hepatotoxicity via suppression of hepatic oxidative stress, pro-inflammation and apoptosis in rats. Toxicol Rep 12:128–134 38304701 10.1016/j.toxrep.2024.01.007 PMC10831491

[CR20] Ali NM, Mamo TT (2023) Pharmacological application of ginger. In Austin Pharmacol Pharm 7(1):1025

[CR21] Ali BH, Blunden G, Tanira MO, Nemmar A (2008) Some phytochemical, pharmacological and toxicological properties of ginger (*Zingiber officinale* Roscoe): a review of recent research. In Food Chem Toxicol 46(2):409–420 10.1016/j.fct.2007.09.085 17950516

[CR22] Ali DA, Abdeen AM, Ismail MF, Mostafa MA (2015) Histological, ultrastructural and immunohistochemical studies on the protective effect of ginger extract against cisplatin-induced nephrotoxicity in male rats. Toxicol Ind Health 31(10):869–880 23552260 10.1177/0748233713483198

[CR23] Alibakhshi T, Khodayar MJ, Khorsandi L, Rashno M, Zeidooni L (2018) Protective effects of zingerone on oxidative stress and inflammation in cisplatin-induced rat nephrotoxicity. Biomed Pharmacother 105:225–232 29857302 10.1016/j.biopha.2018.05.085

[CR24] Almatroodi SA, Alnuqaydan AM, Babiker AY, Almogbel MA, Khan AA, Rahmani AH (2021) 6-Gingerol, a bioactive compound of ginger attenuates renal damage in streptozotocin-induced diabetic rats by regulating the oxidative stress and inflammation. Pharmaceutics 13:317 33670981 10.3390/pharmaceutics13030317 PMC7997342

[CR25] Al-Thubiani WS (2021) The potential protective role of betanin and allicin against adriamycin induced cardiotoxicity in rats. Dissertation, King Abdulaziz University Jeddah

[CR26] Amin A, Hamza AA (2006) Effects of Roselle and ginger on cisplatin-induced reproductive toxicity in rats. Asian J Androl 8(5):607–612 16751998 10.1111/j.1745-7262.2006.00179.x

[CR27] Asuku AO, Ayinla MT, Ajibare AJ, Olajide TS (2024) Mercury chloride causes cognitive impairment, oxidative stress and neuroinflammation in male Wistar rats: the potential protective effect of 6-gingerol-rich fraction of *Zingiber officinale* via regulation of antioxidant defence system and reversal of pro-inflammatory markers increase. Brain Res 1826:148741 38157955 10.1016/j.brainres.2023.148741

[CR28] Auner HW, Cenci S (2015) Recent advances and future directions in targeting the secretory apparatus in multiple myeloma. Br J Haematol 168(1):14–25 25296649 10.1111/bjh.13172

[CR29] Ayustaningwarno F, Anjani G, Ayu AM, Fogliano V (2024) A critical review of ginger’s (Zingiber officinale) antioxidant, anti-inflammatory, and immunomodulatory activities. Front Nutr 11:1364836 38903613 10.3389/fnut.2024.1364836 PMC11187345

[CR30] Baiskhanova D, Schäfer H (2024) The role of Nrf2 in the regulation of mitochondrial function and ferroptosis in pancreatic cancer. Antioxidants 13:696 38929135 10.3390/antiox13060696 PMC11201043

[CR31] Baliga R, Zhang Z, Aliga MB, Ueda N, Shah SV (1998) In vitro and in vivo evidence suggesting a role for iron in cisplatin-induced nephrotoxicity. Kidney Int 53:394–401 9461098 10.1046/j.1523-1755.1998.00767.x

[CR32] Ballester P, Cerdá B, Arcusa R, García-Muñoz AM, Marhuenda J, Zafrilla P (2023) Antioxidant activity in extracts from Zingiberaceae family: cardamom, turmeric, and ginger. Molecules 28(10):4024 37241765 10.3390/molecules28104024 PMC10220638

[CR33] Balogun FO, AdeyeOluwa ET, Ashafa AO (2019) Pharmacological potentials of ginger. In: Wang H (ed) Ginger cultivation and its antimicrobial and pharmacological potentials. IntechOpen London, UK, pp 1–162

[CR34] Baptista Moreno Martin AC, Tomasin R, Luna-Dulcey L, Graminha AE, Araújo Naves M, Teles RH, da Silva VD, da Silva JA, Vieira PC, Annabi B, Cominetti MR (2020) [10]-Gingerol improves doxorubicin anticancer activity and decreases its side effects in triple negative breast cancer models. Cell Oncol 43:915–29 10.1007/s13402-020-00539-z PMC12990714 32761561

[CR35] Bashir N, Ahmad SB, Rehman MU, Muzamil S, Bhat RR, Mir MU, Shazly GA, Ibrahim MA, Elossaily GM, Sherif AY, Kazi M (2021) Zingerone (4-(four-hydroxy-3-methylphenyl) butane-two-1) modulates adjuvant-induced rheumatoid arthritis by regulating inflammatory cytokines and antioxidants. Redox Rep 26(1):62–70 33784959 10.1080/13510002.2021.1907518 PMC8018447

[CR36] Belhadj Slimen I, Najar T, Ghram A, Dabbebi H, Ben Mrad M, Abdrabbah M (2014) Reactive oxygen species, heat stress and oxidative-induced mitochondrial damage. Rev Int J Hyperthermia 30(7):513–523 10.3109/02656736.2014.971446 25354680

[CR37] Berida TI, Adekunle YA, Dada-Adegbola H, Kdimy A, Roy S, Sarker SD (2024) Plant antibacterials: The challenges and opportunities. Heliyon 10:e31145 38803958 10.1016/j.heliyon.2024.e31145 PMC11128932

[CR38] Bhattacharya S (2015) Reactive oxygen species and cellular defense system. In: Rani V, Yadav U (eds) Free Radicals in Human Health and Disease. New Delhi, Springer, pp 17–29

[CR39] Bonsignore G, Martinotti S, Ranzato E (2023) Endoplasmic reticulum stress and cancer: could unfolded protein response be a druggable target for cancer therapy? Int J Mol Sci 24(2):1566 36675080 10.3390/ijms24021566 PMC9865308

[CR40] Bose S, Banerjee S, Mondal A, Chakraborty U, Pumarol J, Croley CR, Bishayee A (2020) Targeting the JAK/STAT signaling pathway using phytocompounds for cancer prevention and therapy. Cells 9(6):1451 32545187 10.3390/cells9061451 PMC7348822

[CR41] Braicu C, Buse M, Busuioc C, Drula R, Gulei D, Raduly L, Rusu A, Irimie A, Atanasov AG, Slaby O, Ionescu C (2019) A comprehensive review on MAPK: a promising therapeutic target in cancer. Cancers 11(10):1618 31652660 10.3390/cancers11101618 PMC6827047

[CR42] Brianna, Lee SH (2023) Chemotherapy: how to reduce its adverse effects while maintaining the potency? Med Oncol 40:88 36735206 10.1007/s12032-023-01954-6

[CR43] Brinkmann V, Fritz G (2022) Prevention of anticancer therapy-induced neurotoxicity: putting DNA damage in perspective. Neurotoxicol 91:1–10 10.1016/j.neuro.2022.04.009 35487345

[CR44] Cao M, Li Y, Famurewa AC, Olatunji OJ (2021) Antidiabetic and nephroprotective effects of polysaccharide extract from the seaweed Caulerpa racemosa in high fructose-streptozotocin induced diabetic nephropathy. Diabetes Metab Syndr Obes: Targets Ther 14:2121–2131 10.2147/DMSO.S302748 PMC8126874 34012278

[CR45] Chakotiya AS, Tanwar A, Narula A, Sharma AK (2017) *Zingiber officinale*: its antibacterial activity on *Pseudomonas aeruginosa* and mode of action evaluated by flow cytometry. Microb Pathog 107:254e260 28389345 10.1016/j.micpath.2017.03.029

[CR46] Chakraborty D, Bishayee K, Ghosh S, Biswas R, Mandal SK, Khuda-Bukhsh AR (2012) [6]-Gingerol induces caspase3 dependent apoptosis and autophagy in cancer cells: drug–DNA interaction and expression of certain signal genes in HeLa cells. Eur J Pharmacol 694:20–29 22939973 10.1016/j.ejphar.2012.08.001

[CR47] Chang JS, Wang KC, Shieh Yeh CF, DE, Chiang LC. (2013) Fresh ginger (*Zingiber officinale*) has anti-viral activity against human respiratory syncytial virus in human respiratory tract cell lines. J Ethnopharmacol 145:146–151 23123794 10.1016/j.jep.2012.10.043

[CR48] Chen CY, Kao CL, Liu CM (2018) The cancer prevention, anti-inflammatory and anti-oxidation of bioactive phytochemicals targeting the TLR4 signalling pathway. Int J Mol Sci 19(9):2729 30213077 10.3390/ijms19092729 PMC6164406

[CR49] Chen C, Gao H, Su X (2021a) Autophagy-related signalling pathways are involved in cancer. Exp Ther Med 22(1):1–3 10.3892/etm.2021.10142 PMC8120650 34007319

[CR50] Chen X, Kang R, Kroemer G, Tang D (2021b) Broadening horizons: the role of ferroptosis in cancer. Nat Rev Clin Oncol 18(5):280–296 33514910 10.1038/s41571-020-00462-0

[CR51] Cheng X, Xu HD, Ran HH, Liang G, Wu FG (2021) Glutathione-depleting nanomedicines for synergistic cancer therapy. ACS Nano 15(5):8039–8068 33974797 10.1021/acsnano.1c00498

[CR52] Chistiakov DA, Shkurat TP, Melnichenko AA, Grechko AV, Orekhov AN (2018) The role of mitochondrial dysfunction in cardiovascular disease: a brief review. Ann Med 50(2):121–127 29237304 10.1080/07853890.2017.1417631

[CR53] Choi J, Ryu J, Bae W, Park A, Nam S, Kim J, Jeong J (2018) Zingerone suppresses tumor development through decreasing Cyclin D1 expression and inducing mitotic arrest. Int J Mol Sci 19:2832 30235818 10.3390/ijms19092832 PMC6163242

[CR54] Chung D, Uddin SMN, Kim J, Kim JK (2015) [6]-Gingerol prevents gamma radiation-induced cell damage in HepG2 cells. J Radioanal Nucl Chem 305:323–328

[CR55] Cocetta V, Ragazzi E, Montopoli M (2019) Mitochondrial involvement in cisplatin resistance. Int J Mol Sci 20(14):3384 31295873 10.3390/ijms20143384 PMC6678541

[CR56] Cocetta V, Tinazzi M, Giacomini I, Rosato B, Ragazzi E, Berretta M, Montopoli M (2023) Clinical evidence of interaction between nutraceutical supplementation and platinum-based chemotherapy. Curr Med Chem 30(19):2141–2164 35638272 10.2174/0929867329666220527120237

[CR57] Cuomo F, Altucci L, Cobellis G (2019) Autophagy function and dysfunction: potential drugs as anti-cancer therapy. Cancers 11(10):1465 31569540 10.3390/cancers11101465 PMC6826381

[CR58] Da Costa R, Passos GF, Quintao NL, Fernandes ES, Maia JR, Campos MM, Calixto JB (2020) Taxane-induced neurotoxicity: pathophysiology and therapeutic perspectives. Br J Pharmacol 177:3127–3146 32352155 10.1111/bph.15086 PMC7312267

[CR59] Davodabadi F, Sajjadi SF, Sarhadi M, Mirghasemi S, Hezaveh MN, Khosravi S, Andani MK, Cordani M, Basiri M, Ghavami S (2023) Cancer chemotherapy resistance: mechanisms and recent breakthrough in targeted drug delivery. Eur J Pharmacol 958:176013 37633322 10.1016/j.ejphar.2023.176013

[CR60] De Freitas Saito R, Rangel MC, Chandler M, Beasock D, Afonin KA, Chammas R (2023) Cancer therapy-induced inflammation and its consequences. In: de Araujo DR, Carneiro-Ramos M (eds) Biotechnology Applied to Inflammatory Diseases: Cellular Mechanisms and Nanomed, 2023rd edn. Springer, Singapore, pp 49–75

[CR61] Deng J, Kohda Y, Chiao H, Wang Y, Hu H, Hewitt SM et al (2001) Interleukin-10 inhibits ischemic and cisplatin-induced acute renal injury. Kidney Int 60:2118–2128 11737586 10.1046/j.1523-1755.2001.00043.x

[CR62] Dobbelstein M, Sørensen CS (2015) Exploiting replicative stress to treat cancer. Nat Rev Drug Discovery 14(6):405–423 25953507 10.1038/nrd4553

[CR63] Dolan RD, McSorley ST, Horgan PG, Laird B, McMillan DC (2017) The role of the systemic inflammatory response in predicting outcomes in patients with advanced inoperable cancer: systematic review and meta-analysis. Crit Rev Oncol Hematol 116:134–146 28693795 10.1016/j.critrevonc.2017.06.002

[CR64] D’Orsi B, Mateyka J, Prehn JH (2017) Control of mitochondrial physiology and cell death by the Bcl-2 family proteins Bax and Bok. Neurochem Int 109:162–170 28315370 10.1016/j.neuint.2017.03.010

[CR65] Dugasani S, Pichika MR, Nadarajah VD, Balijepalli MK, TandraKorlakunta SJN (2010) Comparative antioxidant and anti-inflammatory effects of [6]-gingerol, [8]-gingerol, [10]-gingerol and [6]-shogaol. J Ethnopharmacol 127(2):515–520 19833188 10.1016/j.jep.2009.10.004

[CR66] Dutta A, Hsiao S, Hung C, Chang C, Lin Y, Lin C, Chen T, Huang C (2023) Effect of [6]-gingerol on viral neuraminidase and hemagglutinin-specific T cell immunity in severe influenza. Phytomed Plus 3:100387

[CR67] El-Ashmawy NE, Khedr NF, El-Bahrawy HA, Abo Mansour HE (2018) Ginger extract adjuvant to doxorubicin in mammary carcinoma: study of some molecular mechanisms. Eur J Nutr 57(3):981–989 28229277 10.1007/s00394-017-1382-6

[CR68] El-Bakly WM, Louka ML, El-Halawany AM, Schaalan MF (2012) 6-gingerol ameliorated doxorubicin-induced cardiotoxicity: role of nuclear factor kappa B and protein glycation. Cancer Chemother Pharmacol 70:833–841 23014738 10.1007/s00280-012-1975-y

[CR69] Elfaky MA, Okairy HM, Abdallah HM, Koshak AE, Mohamed GA, Ibrahim SRM, Alzain AA, Hegazy WAH, Khafagy E, Seleem NM (2024) Assessing the antibacterial potential of 6-gingerol: combined experimental and computational approaches. Saudi Pharm J 32:102041 38558886 10.1016/j.jsps.2024.102041 PMC10981156

[CR70] El-Hawwary AA, Omar NM (2019) The influence of ginger administration on cisplatin-induced cardiotoxicity in rat: light and electron microscopic study. Acta Histochem 121(5):553–562 31068261 10.1016/j.acthis.2019.04.013

[CR71] Elseweidy MM, Zaghloul MS, Younis NN (2016) 10-DHGD ameliorates cisplatin-induced nephrotoxicity in rats. Biomed Pharmacother 83:241–246 27376779 10.1016/j.biopha.2016.06.032

[CR72] Elshopakey GE, Almeer R, Alfaraj S, Albasher G, Abdelgawad ME, Abdel Moneim AE, Essawy EA (2022) Zingerone mitigates inflammation, apoptosis and oxidative injuries associated with renal impairment in adriamycin-intoxicated mice. Toxin Reviews 41(3):731–742

[CR73] Essawy AE, Abdel-Wahab WM, Sadek IA, Khamis OM (2018) Dual protective effect of ginger and rosemary extracts against CCl_4_-induced hepatotoxicity in rats. Environ Sci Pollut Res 25:19510–19517 10.1007/s11356-018-2129-5 29730760

[CR74] Famurewa AC, Ufebe OG, Egedigwe CA, Nwankwo OE, Obaje GS (2017) Virgin coconut oil supplementation attenuates acute chemotherapy hepatotoxicity induced by anticancer drug methotrexate via inhibition of oxidative stress in rats. Biomed Pharmacother 87:437–442 28068634 10.1016/j.biopha.2016.12.123

[CR75] Famurewa AC, Folawiyo AM, Enohnyaket EB, Azubuike-Osu SO, Abi I, Obaje SG, Famurewa OA (2018) Beneficial role of virgin coconut oil supplementation against acute methotrexate chemotherapy-induced oxidative toxicity and inflammation in rats. Integr Med Res 7(3):257–263 30271714 10.1016/j.imr.2018.05.001 PMC6160495

[CR76] Famurewa AC, Aja PM, Nwankwo OE, Awoke JN, Maduagwuna EK, Aloke K (2019) *Moringa oleifera* seed oil or virgin coconut oil supplementation abrogates cerebral neurotoxicity induced by antineoplastic agent methotrexate by suppression of oxidative stress and neuroinflammation in rats. J Food Biochem 43(3):e12748 31353570 10.1111/jfbc.12748

[CR77] Famurewa AC, Ekeleme-Egedigwe CA, Onwe CS, Egedigwe UO, Okoro CO, Egedigwe UJ, Asogwa NT (2020a) Ginger juice prevents cisplatin-induced oxidative stress, endocrine imbalance and NO/iNOS/NF-κB signalling via modulating testicular redox-inflammatory mechanism in rats. Andrologia 52(10):1–10. 10.1111/and.13786 10.1111/and.13786 32777091

[CR78] Famurewa AC, Ekeleme-Egedigwe CA, Onwe CS, Egedigwe UO, Okoro CO, Egedigwe UJ, Asogwa NT (2020b) Ginger juice prevents cisplatin-induced oxidative stress, endocrine imbalance and NO/iNOS/NF-κB signalling via modulating testicular redox-inflammatory mechanism in rats. Andrologia 52(10):e13786 32777091 10.1111/and.13786

[CR79] Famurewa AC, Edeogu CO, Offor FI, Besong EE, Akunna GG, Maduagwuna EK (2021) Downregulation of redox imbalance and iNOS/NF-ĸB/caspase-3 signalling with zinc supplementation prevents urotoxicity of cyclophosphamide-induced hemorrhagic cystitis in rats. Life Sci 266(7):118913 33333050 10.1016/j.lfs.2020.118913

[CR80] Famurewa AC, Mukherjee AG, Wanjari UR, Sukumar A, Murali R, Renu K, Vellingiri B, Dey A, Gopalakrishnan AV (2022) Repurposing FDA-approved drugs against the toxicity of platinum-based anticancer drugs. Life Sci 305:120789 35817170 10.1016/j.lfs.2022.120789

[CR81] Famurewa AC, Ekeleme-Egedigwe CA, Ogbu PN, Ajibare AJ, FolawiyoMA Obasi DO, Narayanankutty A (2023) Morin hydrate downregulates inflammation-mediated nitric oxide overproduction and potentiates antioxidant mechanism against anticancer drug doxorubicin oxidative hepatorenal toxicity in rats. Avicenna J Phytomed 13(5):475–487 38089416 10.22038/AJP.2023.22392 PMC10711573

[CR82] Famurewa AC, George MY, Ukwubile CA, Kumar S, Kamal MV, Belle VS, Othman EM, Pai SRK (2024) Trace elements and metal nanoparticles: mechanistic approaches to mitigating chemotherapy-induced toxicity—a review of literature evidence. Biometals. 10.1007/s10534-024-00637-7 39347848 10.1007/s10534-024-00637-7

[CR83] Fang G, Li X, Yang F, Huang T, Qiu C, Peng K, Wang Z, Yang Y, Lan C (2023) Amentoflavone mitigates doxorubicin-induced cardiotoxicity by suppressing cardiomyocyte pyroptosis and inflammation through inhibition of the STING/NLRP3 signalling pathway. Phytomed 117:154922 10.1016/j.phymed.2023.154922 37321078

[CR84] Fekry E, Rahman AA, Awny MM, Makary S (2019) Protective effect of mirtazapine versus ginger against cisplatin-induced testicular damage in adult male albino rats. Ultrastruct Pathol 43(1):66–79 30929557 10.1080/01913123.2019.1592269

[CR85] Foufelle F, Fromenty B (2016) Role of endoplasmic reticulum stress in drug-induced toxicity. Pharmacol Res Perspect 4(1):e00211 26977301 10.1002/prp2.211 PMC4777263

[CR86] Gabr SA, Alghadir AH, Ghoniem GA (2019) Biological activities of ginger against cadmium-induced renal toxicity. Saudi J Biol Sci 26:382–389 10.1016/j.sjbs.2017.08.008 PMC6717148 31485182

[CR87] Gabr SA, Elsaed WM, Eladl MA, Ghoniem GA, El-Sherbiny M, El-Bayoumi KS, Abouhish H, Desouky AM, Abdel-Aziz MM, Eldesoqui M, Elshafey M, Ebrahim HA, Nosseir NS, El-Sayed AMR (2023) Circulating microRNAs as novel biomarkers for measuring the potency of ginger extract against cyclophosphamide toxicity in rat renal tissues: molecular and histopathological study. Eur Rev Med Pharmacol Sci 27(22):10815–10830 38039010 10.26355/eurrev_202311_34448

[CR88] Gadisa DA, Assefa M, Wang SH, Yimer G (2020) Toxicity profile of doxorubicin-cyclophosphamide and doxorubicin-cyclophosphamide followed by paclitaxel regimen and its associated factors among women with breast cancer in Ethiopia: a prospective cohort study. J Oncol Pharm Pract 26(8):1912–1920 32122234 10.1177/1078155220907658

[CR89] Ganaie MA, Al Saeedan A, Madhkali H, Jan BL, Khatlani T, Sheikh IA, Rehman MU, Wani K (2019) Chemopreventive efficacy zingerone (4-[4-hydroxy-3-methylphenyl] butan-2-one) in experimental colon carcinogenesis in Wistar rats. Environ Toxicol 34:610–625 30720227 10.1002/tox.22727

[CR90] Gavanji S, Bakhtari A, Famurewa AC, Othman EM (2023) Cytotoxic activity of herbal medicines as assessed in vitro: a review. Chem Biodivers 20:e202201098 36595710 10.1002/cbdv.202201098

[CR91] Gawel K, Kukula-Koch W, Banono NS, Nieoczym D, Targowska-Duda KM, Czernicka L, Parada-Turska J, Esguerra CV (2021) 6-Gingerol, a major constituent of *Zingiber officinale* Rhizoma, exerts anticonvulsant activity in the pentylenetetrazole-induced seizure model in larval Zebrafish. Int J Mol Sci 22:7745 34299361 10.3390/ijms22147745 PMC8305044

[CR92] Ghayur MN, Gilani AH (2005) Ginger lowers blood pressure through blockade of voltage-dependent calcium channels. J Cardiovasc Pharmacol 45(1):74–80 15613983 10.1097/00005344-200501000-00013

[CR93] Guan X, Wang Y, Li W, Liu X, Jiang J, Bian W, Xu C, Sun Y, Zhang C (2023) The effects and mechanism of LncRNA NORAD on doxorubicin-induced cardiotoxicity. Toxicology 494:153587 37406984 10.1016/j.tox.2023.153587

[CR94] Gwon MG, Gu H, Leem J, Park KK (2021) Protective effects of 6-shogaol, an active compound of ginger, in a murine model of cisplatin-induced acute kidney injury. Molecules 26:5931 34641472 10.3390/molecules26195931 PMC8512008

[CR95] Hamed MA, Adegboyega OO, Ojo OI, Akhigbe TM, Fajuyitan FD, Adeyemo OC, Odebunmi TF, Adeniyi OS, Omole IA, Akhigbe RE (2024) Glutamine-mediated modulation of XO/uric acid/NF-kB signaling pathway ameliorates intestinal I/R-induced bacterial translocation and Cardiorenal inflammatory injury. Cell Biochem Biophys 82(2):1007–1018 10.1007/s12013-024-01252-6 38530591

[CR96] Hasan S, Danishuddin M, Khan AU (2015) Inhibitory effect of *Zingiber officinale* towards *Streptococcus mutans* virulence and caries development: *in vitro* and *in vivo* studies. BMC Microbiol 15(1):1–14 25591663 10.1186/s12866-014-0320-5 PMC4316655

[CR97] Haybar H, Bandar B, Torfi E, Mohebbi A, Saki N (2023) Cytokines and their role in cardiovascular diseases. Cytokine 169:156261 37413877 10.1016/j.cyto.2023.156261

[CR98] He L, Shi Y (2023) Reduced glutathione ameliorates acute kidney injury by inhibiting ferroptosis. Mol Med Rep 27:123 37203404 10.3892/mmr.2023.13011 PMC10206672

[CR99] He L, He T, Farrar S, Ji L, Liu T, Ma X (2017) Antioxidants maintain cellular redox homeostasis by elimination of reactive oxygen species. Cell Physiol Biochem 44(2):532–553 29145191 10.1159/000485089

[CR100] He Y, Xi J, Fang J, Zhang B, Cai W (2023) Aloe-emodin alleviates doxorubicin-induced cardiotoxicity via inhibition of ferroptosis. Free Radical Biol Med 206:13–21 37364691 10.1016/j.freeradbiomed.2023.06.025

[CR101] Hilton BA, Li Z, Musich PR, Wang H, Cartwright BM, Serrano M, Zhou XZ, Lu KP, Zou Y (2015) ATR plays a direct antiapoptotic role at mitochondria, which is regulated by prolyl isomerase Pin1. Mol Cell 60(1):35–46 26387736 10.1016/j.molcel.2015.08.008 PMC4592481

[CR102] Hosseini A, Alavi MS, Toos MG, Jamialahmadi T, Sahebka A (2024) 6-Gingerol, an ingredient of Zingiber officinale, abrogates lipopolysaccharide-induced cardiomyocyte injury by reducing oxidative stress and inflammation. J Agric Food Res 15:101034

[CR103] Hosseinzadeh A, Juybari BK, Fatemi MJ, Kamarul T, Bagheri A, Tekiyehmaroof N, Sharifi AM (2017) Protective effect of ginger (*Zingiber officinale* Roscoe) extract against oxidative stress and mitochondrial apoptosis induced by interleukin-1β in cultured chondrocytes. Cells Tissues Organs 204(5–6):241–250 28877520 10.1159/000479789

[CR104] Hu Z, Zhang H, Yi B, Yang S, Liu J, Hu J, Wang J, Cao K, Zhang W (2020) VDR activation attenuate cisplatin induced AKI by inhibiting ferroptosis. Cell Death Dis 11:73 31996668 10.1038/s41419-020-2256-z PMC6989512

[CR105] Hu J, Gu W, Ma N, Fan X, Ci X (2022a) Leonurine alleviates ferroptosis in cisplatin-induced acute kidney injury by activating the Nrf2 signalling pathway. Br J Pharmacol 179:3991–4009 35303762 10.1111/bph.15834

[CR106] Hu Q, Wei W, Wu D, Huang F, Li M, Li W, Yin J, Peng Y, Lu Y, Zhao Q, Liu L (2022b) Blockade of GCH1/BH4 axis activates ferritinophagy to mitigate the resistance of colorectal cancer to erastin-induced ferroptosis. Front Cell Dev Biol 10:810327 35223839 10.3389/fcell.2022.810327 PMC8866854

[CR107] Hu S, Zhao H (2021) 6-gingerol promotes g2/m phase cell cycle arrest and apoptosis in hepatocellular carcinoma. Acta Poloniae Pharmaceutica 78(1):33–40

[CR108] Ikeda Y, Hamano H, Horinouchi Y, Miyamoto L, Hirayama T, Nagasawa H, Tamaki T, Tsuchiya K (2021) Role of ferroptosis in cisplatin-induced acute nephrotoxicity in mice. J Trace Element Med Biol 67:126798 10.1016/j.jtemb.2021.126798 34087581

[CR109] Jan R (2019) Understanding apoptosis and apoptotic pathways targeted cancer therapeutics. Adv Pharm Bull 9(2):205 31380246 10.15171/apb.2019.024 PMC6664112

[CR110] Jeong C, Bode AM, Pugliese A, Cho Y, Kim H, Shim J, Jeon Y, Li H, Jiang H, Dong Z (2009) [6]-Gingerol suppresses colon cancer growth by targeting leukotriene A4 hydrolase. Cancer Res 69(13):5584–5591 19531649 10.1158/0008-5472.CAN-09-0491

[CR111] Jezek P, Hlavata L (2005) Mitochondria in homeostasis of reactive oxygen species in cell, tissues, and organism. Int J Biochem Cell Biol 37:2478–2503 16103002 10.1016/j.biocel.2005.05.013

[CR112] Jian B, Pang J, Xiong H, Zhang W, Zhan T, Su Z, Lin H, Zhang H, He W, Zheng Y (2021) Autophagy-dependent ferroptosis contributes to cisplatin-induced hearing loss. Toxicol Lett 350:249–260 34302894 10.1016/j.toxlet.2021.07.010

[CR113] Jiang Y, Li Z, Ma Q, Dong W, Yao Q, Yu D (2023) Aucubin protects mouse cochlear hair cells from cisplatin-induced ototoxicity via activation of the PI3K/AKT/STAT3 pathway. Biochem Pharmacol 209:115440 36720354 10.1016/j.bcp.2023.115440

[CR114] Kandemir FM, Mahamadu A (2021) Investigation of the effect of zingerone on some biochemical parameters on cisplatin-induced liver toxicity in rats. Kocatepe Vet J 14(3):325–338

[CR115] Kang DY, Park S, Song KS, Bae SW, Lee J, Jang K, Park Y (2023) anticancer effects of 6-gingerol through downregulating iron transport and PD-L1 expression in non-small cell lung cancer cells. Cells 12:2628 37998363 10.3390/cells12222628 PMC10670414

[CR116] Karampour NS, Arzi A, Rezaie A, Pashmforoosh M, Kordi F (2019) Gastroprotective effect of zingerone on ethanol-induced gastric ulcers in rats. Medicina 55:0064 10.3390/medicina55030064 PMC6473471 30862060

[CR117] Karimian A, Ahmadi Y, Yousefi B (2016) Multiple functions of p21 in cell cycle, apoptosis and transcriptional regulation after DNA damage. DNA Repair 42:63–71 27156098 10.1016/j.dnarep.2016.04.008

[CR118] Kaufmann HE (2008) Paul Ehrlich: founder of chemotherapy. Nat Rev Drug Discovery 7:373 18456958 10.1038/nrd2582

[CR119] Kawano I, Adamcova M (2022) MicroRNAs in doxorubicin-induced cardiotoxicity: The DNA damage response. Front Pharmacol 13:1055911 36479202 10.3389/fphar.2022.1055911 PMC9720152

[CR120] Khan T, Waseem R, Zehra Z, Aiman A, Bhardwaj P, Ansari J, Hassan MI, Islam A (2022) Mitochondrial dysfunction: pathophysiology and mitochondria-targeted drug delivery approaches. Pharmaceutics 14(12):2657 36559149 10.3390/pharmaceutics14122657 PMC9785072

[CR121] Khandia R, Dadar M, Munjal A, Dhama K, Karthik K, Tiwari R, Yatoo MI, Iqbal HM, Singh KP, Joshi SK, Chaicumpa W (2019) A comprehensive review of autophagy and its various roles in infectious, non-infectious, and lifestyle diseases: current knowledge and prospects for disease prevention, novel drug design, and therapy. Cells 8(7):674 31277291 10.3390/cells8070674 PMC6678135

[CR122] Kim H, Park H (2013) Ginger extract inhibits biofilm formation by *Pseudomonas aeruginosa* PA14. PLoS ONE 8(90):e76106 24086697 10.1371/journal.pone.0076106 PMC3785436

[CR123] Kim S, Gang J, Lee JH, Yang H, Cheon C, Ko SG, Bae H, Kim W (2022) [6]-shogaol attenuates oxaliplatin-induced allodynia through serotonergic receptors and GABA in the spinal cord in mice. Pharmaceuticals (Basel) 15(6):726 35745645 10.3390/ph15060726 PMC9227032

[CR124] Kim H, Park K-T, Jo H, Shin Y, Chung G, Ko S-G, Jin Y-H, Kim W (2023) The effect of ginger extract on cisplatin-induced acute anorexia in rats. Front Pharmacol 14:1267254 10.3389/fphar.2023.1267254 PMC10665510 38026983

[CR125] Kongsui R, Jittiwat J (2023) Ameliorative effects of 6-gingerol in cerebral ischemia are mediated via the activation of antioxidant and anti-inflammatory pathways. Biomed Rep 18(26):1–10 36909941 10.3892/br.2023.1608 PMC9996095

[CR126] Kucukler S, Darendelioğlu E, Caglayan C, Ayna A, Yıldırım S, Kandemir FM (2020) Zingerone attenuates vancomycin-induced hepatotoxicity in rats through regulation of oxidative stress, inflammation and apoptosis. Life Sci 259:118382 32898532 10.1016/j.lfs.2020.118382

[CR127] Kumar L, Chhibber S, Harjai K (2013) Zingerone inhibit biofilm formation and improve antibiofilm efficacy of ciprofloxacin against *Pseudomonas aeruginosa* PAO1. Fitoterapia 90:73–78 23831483 10.1016/j.fitote.2013.06.017

[CR128] Kumar L, Chhibber S, Kumar R, Kumar M, Harjai K (2015) Zingerone silences quorum sensing and attenuates virulence of *Pseudomonas aeruginosa*. Fitoterapia 102:84–95 25704369 10.1016/j.fitote.2015.02.002

[CR129] Kun E, Tsang YT, Ng CW, Gershenson DM, Wong KK (2021) MEK inhibitor resistance mechanisms and recent developments in combination trials. Cancer Treat Rev 92:102137 33340965 10.1016/j.ctrv.2020.102137

[CR130] Lee HS, Seo EY, Kang NE, Kim WK (2008) [6]-Gingerol inhibits metastasis of MDA-MB-231 human breast cancer cells. J Nutr Biochem 19(5):313–319 17683926 10.1016/j.jnutbio.2007.05.008

[CR131] Lee I, Kim S, Nagar H, Choi SJ, Jeon BH, Piao S, Kim CS (2020) CR6-interacting factor 1 deficiency reduces endothelial nitric oxide synthase activity by inhibiting biosynthesis of tetrahydrobiopterin. Sci Rep 10(1):842 31964986 10.1038/s41598-020-57673-9 PMC6972730

[CR132] Lee JH, Min D, Lee D, Kim W (2021) *Zingiber officinale* roscoe rhizomes attenuate oxaliplatin-induced neuropathic pain in mice. Molecules 26(3):548 33494465 10.3390/molecules26030548 PMC7866215

[CR133] Li G, Ding K, Qiao Y, Zhang L, Zheng L, Pan T, Zhang L (2020a) Flavonoids regulate inflammation and oxidative stress in cancer. Molecules 25(23):5628 33265939 10.3390/molecules25235628 PMC7729519

[CR134] Li J, Cao F, Yin HL, Huang ZJ, Lin ZT, Mao N, Sun B, Wang G (2020b) Ferroptosis: past, present and future. Cell Death Di 11(2):88 10.1038/s41419-020-2298-2 PMC6997353 32015325

[CR135] Li Y, Xu B, Ren X et al (2022) Inhibition of CISD2 promotes ferroptosis through ferritinophagy-mediated ferritin turnover and regulation of p62-Keap1-NRF2 pathway. Cell Mol Biol Lett 27(1):81 36180832 10.1186/s11658-022-00383-z PMC9523958

[CR136] Li Y, Li K, Zhao W, Wang H, Xue X, Chen X, Li W, Xu P, Wang K, Liu P, Tian X, Fu R (2023) VPA improves ferroptosis in tubular epithelial cells after cisplatin-induced acute kidney injury. Front Pharmacol 14:1147772. 10.3389/fphar.2023.1147772 37153759 10.3389/fphar.2023.1147772 PMC10155836

[CR137] Li W, Zhang Y, Wang Q, Wang Y, Fan Y, Shang E, Jiang S, Duan J (2024) 6-Gingerol ameliorates ulcerative colitis by inhibiting ferroptosis based on the integrative analysis of plasma metabolomics and network pharmacology. Food Funct 15:6054 38753306 10.1039/d4fo00952e

[CR138] Lin J, Zhang Y, Guan H, Li S, Sui Y, Hong L, Zheng Z, Huang M (2024) Myricitrin inhibited ferritinophagy-mediated ferroptosis in cisplatin-induced human renal tubular epithelial cell injury. Front Pharmacol 15:1372094 38910888 10.3389/fphar.2024.1372094 PMC11190325

[CR139] Liu Y, Zeng L, Yang Y, Chen C, Wang D, Wang H (2020) Acyl-CoA thioesterase 1 prevents cardiomyocytes from doxorubicin-induced ferroptosis via shaping the lipid composition. Cell Death Dis 11:756 32934217 10.1038/s41419-020-02948-2 PMC7492260

[CR140] Liu B, Zhou H, Tan L, Siu KT, Guan X-Y (2024) Exploring treatment options in cancer: tumor treatment strategies. Signal Transduct Target Ther 9:175 39013849 10.1038/s41392-024-01856-7 PMC11252281

[CR141] Lomeli N, Lepe J, Gupta K, Bota DA (2021) Cognitive complications of cancer and cancer-related treatments- Novel paradigms. Neurosci Lett 749:135720 33582187 10.1016/j.neulet.2021.135720 PMC8423125

[CR142] Luo J, Chen J, Yang C, Tan J, Zhao J, Jiang N, Zhao Y (2021) 6-Gingerol protects against cerebral ischemia/reperfusion injury by inhibiting NLRP3 inflammasome and apoptosis via TRPV1 / FAF1 complex dissociation-mediated autophagy Jing. Int Immunopharmacol 100:108146 34537481 10.1016/j.intimp.2021.108146

[CR143] Lv X, Wang M, Qin Q, Lu P, Qin G (2021) 6-Gingerol relieves myocardial ischaemia/reperfusion injury by regulating lncRNA H19/miR-143/ATG7 signaling axis-mediated autophagy. Lab Invest 101:865–877 33758383 10.1038/s41374-021-00575-9

[CR144] Ma SQ, Guo Z, Liu FY, Hasan SG, Yang D, Tang N, Tang QZ (2021) 6- Gingerol protects against cardiac remodeling by inhibiting the p38 mitogen-activated protein kinase pathway. Acta Pharmacologica Sinica 42(10):1575–1586 33462378 10.1038/s41401-020-00587-z PMC8463710

[CR145] Ma TL, Chen JX, Zhu P, Zhang CB, Zhou Y, Duan JX (2022) Focus on ferroptosis regulation: exploring novel mechanisms and applications of ferroptosis regulator. Life Sci 307:120868–120881 35940216 10.1016/j.lfs.2022.120868

[CR146] Malik A, Bagchi AK, Jassal DS, Singal PK (2022) Interleukin-10 mitigates doxorubicin-induced endoplasmic reticulum stress as well as cardiomyopathy. Biomed 10:890 10.3390/biomedicines10040890 PMC9027958 35453640

[CR147] Mao QQ, Xu XY, Cao SY, Gan RY, Corke H, Beta T, Bin LH (2019) Bioactive compounds and bioactivities of ginger (*Zingiber officinale* Roscoe). Foods 8(6):185 31151279 10.3390/foods8060185 PMC6616534

[CR148] Mapuskar KA, Steinbach EJ, Zaher A, Riley DP, Beardsley RA, Keene JL, Holmlund JT, Anderson CM, Zepeda-Orozco D, Buatti JM, Spitz DR (2021) Mitochondrial superoxide dismutase in cisplatin-induced kidney injury. Antioxidants 10(9):1329 34572961 10.3390/antiox10091329 PMC8469643

[CR149] Marin GE, Neag MA, Burlacu CC, Buzoianu AD (2022) The protective effects of nutraceutical components in methotrexate-induced toxicity models-an overview. Microorganisms 10:2053 36296329 10.3390/microorganisms10102053 PMC9608860

[CR150] Mehrzadi S, Khalili H, Fatemi I, Malayeri A, Siahpoosh A, Goudarzi M (2021) Zingerone mitigates carrageenan-induced inflammation through antioxidant and anti-inflammatory activities. Inflammation 44:186–193 32803664 10.1007/s10753-020-01320-y

[CR151] Mir BA, Amin I, Rehman MU, Razak R, Ali A, Baba OK, Fatima B, Ali R, Bilal S, Muzamil S, Hussain I (2018) Chemoprotective potential of zingerone (vanillyl acetone) in cyclophosphamide-induced hepatic toxicity. Pharmacogn Mag 14(57s):s434–s439

[CR152] Mishra AP, Salehi B, Sharifi-Rad M, Pezzani R, Kobarfard F, Sharifi-Rad J, Nigam M (2018) Programmed cell death, from a cancer perspective: an overview. Mol Diagn Ther 22:281–295 29560608 10.1007/s40291-018-0329-9

[CR153] Mortezaee K, Najafi M, Farhood B, Ahmadi A, Shabeeb D, Musa AE (2019) NF-κB targeting for overcoming tumor resistance and normal tissues toxicity. J Cell Physiol 234(10):17187–17204 30912132 10.1002/jcp.28504

[CR154] Mukkavilli R, Gundala SR, Yang C, Donthamsetty S, Cantuaria G, Jadhav GR, Vangala S, Reid MD, Aneja R (2014) Modulation of cytochrome P450 metabolism and transport across intestinal epithelial barrier by ginger biophenolics. PLoS ONE 9(9):e108386 25251219 10.1371/journal.pone.0108386 PMC4177392

[CR155] Musolino C, Allegra A, Innao V, Allegra AG, Pioggia G, Gangemi S (2017) Inflammatory and anti-inflammatory equilibrium, proliferative and antiproliferative balance: the role of cytokines in multiple myeloma. Mediators Inflamm 2017(1):1852517 29089667 10.1155/2017/1852517 PMC5635476

[CR156] Nagoor Meeran MF, Arunachalam S, Azimullah S, Saraswathiamma D, Albawardi A, Almarzooqi S, Jha NK, Subramanya S, Beiram R, Ojha S (2023) α-Bisabolol, a dietary sesquiterpene, attenuates doxorubicin-induced acute cardiotoxicity in rats by inhibiting cellular signaling pathways, Nrf2/Keap-1/HO-1, Akt/mTOR/GSK-3β, NF-κB/p38/MAPK, and NLRP3 inflammasomes regulating oxidative stress and inflammatory cascades. Int J Mol Sci 24:14013 37762315 10.3390/ijms241814013 PMC10530367

[CR157] Neuzil J, Pervaiz S, Fulda S (eds) (2014) Mitochondria: the anti-cancer target for the third millennium. Springer, The Netherlands

[CR158] Niazvand F, Ashtari A, Chamkouri N, Azari M (2023) Hepatoprotective effects of Alpinia officinarum rhizome extract on cisplatin-induced hepatotoxicity in rats: a biochemical, histopathological and immunohistochemical study. J Trace Elem Med Biol 80:127306 37757646 10.1016/j.jtemb.2023.127306

[CR159] Okkay IF, Famurewa AC, Bayram C, Okkay U, Mendil AS, Sezen S, Ayaz T, Gecili I, Ozkaraca M, Senyayla S, Hacimuftuoglu A (2024) Arbutin abrogates cisplatin-induced hepatotoxicity via upregulating Nrf2/HO-1 and suppressing genotoxicity, NF-κB/iNOS/TNF-α and caspase-3/Bax/Bcl2 signaling pathways in rats. Toxicol Res. 10.1093/toxres/tfae075 10.1093/toxres/tfae075 PMC11102346 38770183

[CR160] Ou W, Liu H, Chen C, Yang C, Zhao X, Zhang Y, Zhang Z, Huang S, Mo H, Lu W, Wang X, Chen A, Yan J, Song X (2024) Spexin inhibits excessive autophagy-induced ferroptosis to alleviate doxorubicin-induced cardiotoxicity by upregulating Beclin 1. Br J Pharmacol 181:4195–4213 10.1111/bph.16484 38961632

[CR161] Ouyang Z-Q, Shao L-S, Wang W, Ke T, Chen D, Zheng G, Duan X, Chu J, Zhu Y, Yang L, Shan H, Huang L, Liao C (2023) Low intensity pulsed ultrasound ameliorates adriamycin-induced chronic renal injury by inhibiting ferroptosis. Redox Rep 28(1):2251237 37652897 10.1080/13510002.2023.2251237 PMC10472869

[CR162] Pan Q, Liu P, Wan M (2023) 6-Gingerol attenuates sepsis-induced acute lung injury by suppressing NLRP3 inflammasome through Nrf2 activation. Folia Histochem Cytobiol 61(1):68–80 36734635 10.5603/FHC.a2023.0002

[CR163] Park YJ, Wen J, Bang S, Park SW, Song SY (2006) [6]-Gingerol induces cell cycle arrest and cell death of mutant p53-expressing pancreatic cancer cells. Yonsei Med J 47(5):688–697 17066513 10.3349/ymj.2006.47.5.688 PMC2687755

[CR164] Park KT, Jo H, Kim B, Kim W (2023) Red ginger extract prevents the development of oxaliplatin-induced neuropathic pain by inhibiting the spinal noradrenergic system in mice. Biomedicines 11:432 36830967 10.3390/biomedicines11020432 PMC9953630

[CR165] Pfeffer CM, Singh AT (2018) Apoptosis: a target for anticancer therapy. Int J Mol Sci 19(2):448 29393886 10.3390/ijms19020448 PMC5855670

[CR166] Pucci C, Martinelli C, Ciofani G (2019) Innovative approaches for cancer treatment: current perspectives and new challenges. ecancer 13:961 10.3332/ecancer.2019.961 PMC6753017 31537986

[CR167] Qian S, Fang H, Zheng L, Liu M (2021) Zingerone suppresses cell proliferation via inducing cellular apoptosis and inhibition of the PI3K/AKT/mTOR signaling pathway in human prostate cancer PC-3 cells. J Biochem Mol Toxicol 35:e22611 32905641 10.1002/jbt.22611

[CR168] Ramos-Casals M, Brahmer JR, Callahan MK, Flores-Chávez A, Keegan N, Khamashta MA, Lambotte O, Mariette X, Prat A, Suárez-Almazor ME (2020) Immune-related adverse events of checkpoint inhibitors. Nat Rev Dis Primers 6(1):38 32382051 10.1038/s41572-020-0160-6 PMC9728094

[CR169] Rao BN, Archana PR, Aithal BK, Rao BSS (2011) Protective effect of Zingerone, a dietary compound against radiation induced genetic damage and apoptosis in human lymphocytes. Eur J Pharmacol 657:59–66 21335001 10.1016/j.ejphar.2011.02.002

[CR170] Rastogi N, Duggal S, Singh SK, Porwal K, Srivastava VK, Maurya R, Bhatt MLB, Mishra DP (2015) Proteasome inhibition mediates p53 reactivation and anticancer activity of 6-Gingerol in cervical cancer cells. Oncotarget 6(41):43310–43325 26621832 10.18632/oncotarget.6383 PMC4791234

[CR171] Razali N, Dewa A, Asmawi MZ, Mohamed N, Manshor NM (2020) Mechanisms underlying the vascular relaxation activities of *Zingiber officinale* var. rubrum in thoracic aorta of spontaneously hypertensive rats. J Integr Med 18(1):46–58 31882255 10.1016/j.joim.2019.12.003

[CR172] Rezatabar S, Karimian A, Rameshknia V, Parsian H, Majidinia M, Kopi TA, Bishayee A, Sadeghinia A, Yousefi M, Monirialamdari M, Yousefi B (2019) RAS/MAPK signaling functions in oxidative stress, DNA damage response and cancer progression. J Cell Physiol 234(9):14951–14965 30811039 10.1002/jcp.28334

[CR173] Rocca C, De Francesco EM, Pasqua T, Granieri MC, De Bartolo A, Gallo Cantafio ME, Muoio MG, Gentile M, Neri A, Angelone T, Viglietto G (2022) Mitochondrial determinants of anti-cancer drug-induced cardiotoxicity. Biomedicines 10(3):520 35327322 10.3390/biomedicines10030520 PMC8945454

[CR174] Romero A, Forero M, Sequeda-Castañeda LG, Grismaldo A, Iglesias J, Celis-Zambrano CA, Schuler I, Morales L (2018) Effect of ginger extract on membrane potential changes and AKT activation on a peroxide-induced oxidative stress cell model. J King Saud Univ Sci 30(2):263–269

[CR175] Sahoo BM, Banik BK, Borah P, Jain A (2022) Reactive oxygen species (ROS): key components in cancer therapies. Anti-Cancer Agents Med Chem (Formerly Curr Med Chem-Anti-Cancer Agents) 22(2):215–22 10.2174/1871520621666210608095512 34102991

[CR176] Sahu BD, Kalvala AK, Koneru M, Mahesh Kumar J, Kuncha M, Rachamalla SS, Sistla R (2014) Ameliorative effect of fisetin on cisplatin-induced nephrotoxicity in rats via modulation of NF-κB activation and antioxidant defence. PLoS ONE 9(9):e105070 25184746 10.1371/journal.pone.0105070 PMC4153571

[CR177] Seufferlein T, Porzner M, Heinemann V, Tannapfel A, Stuschke M, Uhl W (2014) Ductal Pancreatic Adenocarcinoma. Dtsch Arztebl Int 111:396–402 24980565 10.3238/arztebl.2014.0396 PMC4078228

[CR178] Seungyoon BY, Pekkurnaz G (2018) Mechanisms orchestrating mitochondrial dynamics for energy homeostasis. J Mol Biol 430(21):3922–3941 30089235 10.1016/j.jmb.2018.07.027 PMC6186503

[CR179] Shamsabadi S, Nazer Y, Ghasemi J, Mahzoon E, Rahimi VB, Ajiboye BO, Askari VR (2023) Promising influences of zingerone against natural and chemical toxins: a comprehensive and mechanistic review. Toxicon 233:107247 10.1016/j.toxicon.2023.107247 37562703

[CR180] Sim AA, Cheam JY, Law JW, Letchumanan V, Kumari Y, Ogawa S, Wong SH, Chan KG, Tan LT (2023) The ameliorative role of probiotics in 5-fluorouracil induced intestinal mucositis. Prog Microbes Mol Biol 6(1):a0000339

[CR181] Soliman AF, Anees LM, Ibrahim DM (2018) Cardioprotective effect of zingerone against oxidative stress, inflammation, and apoptosis induced by cisplatin or gamma radiation in rats. Naunyn Schmiedebergs Arch Pharmacol 391:819–832 29736620 10.1007/s00210-018-1506-4

[CR182] Song J, Fan H, Li H, Ding H, Lv Q, Hou S (2016) Zingerone ameliorates lipopolysaccharide-induced acute kidney injury by inhibiting Toll-like receptor 4 signaling pathway. Eur J Pharmacol 772:108–114 26698392 10.1016/j.ejphar.2015.12.027

[CR183] Song W, Zhang L, Cui X, Wang R, Ma J, Xu Y, Jin Y, Wang D, Lu Z (2024a) Nobiletin alleviates cisplatin-induced ototoxicity via activating autophagy and inhibiting NRF2/GPX4-mediated ferroptosis. Sci Rep 14:7889 38570541 10.1038/s41598-024-55614-4 PMC10991266

[CR184] Song Z, Li Z, Pan T, Liu T, Gong B, Wang Z, Liu K, Fan H (2024b) Protopanaxadiol prevents cisplatin-induced acute kidney injury by regulating ferroptosis. J Pharm Pharmacol 1–13 10.1093/jpp/rgae050 10.1093/jpp/rgae050 38708970

[CR185] Srinivasan K (2014) Antioxidant potential of spices and their active constituents. Crit Rev Food Sci Nutr 54(3):352–372 24188307 10.1080/10408398.2011.585525

[CR186] Srinivasan K, Adhya P, Sharma SS (2019) Nutraceutical potential of ginger. In: Gupta R, Srivastava A, Lall R (eds) Nutraceuticals in Veterinary Medicine. Springer, Cham pp 51–70

[CR187] Sun M, Chang H, Jiang F, Zhang W, Yang Q, Wang X, Lv G, Lin H, Luo H, Lin Z et al (2024) Hazel leaf polyphenol extract alleviated cisplatin-induced acute kidney injury by reducing ferroptosis through inhibiting hippo signalling. Molecules 29:1729 38675549 10.3390/molecules29081729 PMC11051766

[CR188] Tadagavadi RK, Reeves WB (2010) Endogenous IL-10 attenuates cisplatin nephrotoxicity: role of dendritic cells. J Immunol 185(8):4904–4911 20844196 10.4049/jimmunol.1000383 PMC3169908

[CR189] Tadokoro T, Ikeda M, Ide T, Deguchi H, Ikeda S, Okabe K, Ishikita A, Matsushima S, Koumura T, Yamada KI, Imai H, Tsutsui H (2020) Mitochondria-dependent ferroptosis plays a pivotal role in doxorubicin cardiotoxicity. JCI Insight 5(9):1–20 10.1172/jci.insight.132747 PMC7253028 32376803

[CR190] Tahir AA, Sani NFA, Murad NA, Makpol S, Ngah WZW, Yusof YAM (2015) Combined ginger extract & Gelam honey modulate Ras/ERK and PI3K/AKT pathway genes in colon cancer HT29 cells. Nutr J 14(1):31 25889965 10.1186/s12937-015-0015-2 PMC4390091

[CR191] Tahoun E, Elgedawy G, El-Bahrawy A (2021) Cytoprotective effect of ginger extract on cisplatin-induced hepatorenal toxicity in rats via modulation of oxidative stress, inflammation and apoptosis: histopathological, biochemical and immunohistochemical study. Comp Clin Pathol 30(4):647–663

[CR192] Thurston DE (2007) Introduction to cancer In: Chemistry and Pharmacology of anticancer drugs, 1st edn. Taylor and Francis Group, New York, p 62

[CR193] Tsai Y, Xia C, Sun Z (2020) The Inhibitory effect of 6-gingerol on ubiquitin-specific peptidase 14 enhances autophagy-dependent ferroptosis and anti-tumor in vivo and in vitro. Front Pharmacol 11:598555 33281606 10.3389/fphar.2020.598555 PMC7691590

[CR194] Ueno N, Hasebe T, Kaneko A, Yamamoto M, Fujiya M, Kohgo Y, Kono T, Wang C, Yuan C, Bissonnette M, Chang EB, Musch MW (2014) TU-100 (Daikenchuto) and ginger ameliorate anti-CD3 antibody induced T cell-mediated murine enteritis: Microbe-independent effects involving Akt and NF-kB suppression. PLoS ONE 9(5):e97456 24857966 10.1371/journal.pone.0097456 PMC4032249

[CR195] Vera M, Barahona MJ, Nova-Lamperti E, Nualart F, Ferrada L (2024) The phenol red compound: a potential artifact in pharmacological induction of ferroptosis. Free Rad Biol Med. 10.1016/j.freeradbiomed.2024.06.023 38944214 10.1016/j.freeradbiomed.2024.06.023

[CR196] Vogel CF, Van Winkle LS, Esser C, Haarmann-Stemmann T (2020) The aryl hydrocarbon receptor as a target of environmental stressors–Implications for pollution mediated stress and inflammatory responses. Redox Biol 34:101530 32354640 10.1016/j.redox.2020.101530 PMC7327980

[CR197] Wang Y, Yi Y (2024) CISD2 downregulation participates in the ferroptosis process of human ovarian SKOV-3 cells through modulating the wild type p53-mediated GLS2/SAT1/SLC7A11 and Gpx4/TRF signaling pathwayCISD2 silence involves in ferroptosis. Tissue Cell. 10.1016/j.tice.2024.102458 10.1016/j.tice.2024.102458 38991271

[CR198] Wang W, Li CY, Wen XD, Li P, Qi LW (2009) Simultaneous determination of 6-gingerol, 8-gingerol, 10-gingerol and 6-shogaol in rat plasma by liquid chromatography-mass spectrometry: Application to pharmacokinetics. J Chromatogr B Analyt Technol Biomed Life Sci 877(8–9):671–679 19201263 10.1016/j.jchromb.2009.01.021

[CR199] Wang J, Shao H, Zhang Y, Ge T, Chen X, Mou X (2024a) Traditional chinese medicine as a protective strategy against chemotherapy-induced cardiotoxicity: an overview of the literature. J Trad Compliment Med. 10.1016/j.jtcme.2024.06.010 10.1016/j.jtcme.2024.06.010 PMC11883632 40060147

[CR200] Wang X, Ren X, Lin X, Li Q, Zhang Y, Deng J, Chen B, Ru G, Luo Y, Lin N (2024b) Recent progress of ferroptosis in cancers and drug discovery. Asian J Pharm Sci 100939. 10.1016/j.ajps.2024.100939 10.1016/j.ajps.2024.100939 PMC11378902 39246507

[CR201] Waseem M, Wang BD (2023) Promising strategy of mPTP modulation in cancer therapy: an emerging progress and future insight. Int J Mol Sci 24(6):5564 36982637 10.3390/ijms24065564 PMC10051994

[CR202] Wen J, Wang J, Li P, Wang R, Wang J, Zhou X, Zhang L, Li H, Wei S, Cai H, Zhao Y (2019) Protective effects of higenamine combined with [6]-gingerol against doxorubicin-induced mitochondrial dysfunction and toxicity in H9c2 cells and potential mechanisms. Biomed Pharmacother 115:108881 31028997 10.1016/j.biopha.2019.108881

[CR203] Weng CJ, Wu CF, Huang HW, Ho CT, Yen GC (2010) Anti-invasion effects of 6-shogaol and 6-gingerol, two active components in ginger, on human hepatocarcinoma cells. Mol Nutr Food Res 54(11):1618–1627 20521273 10.1002/mnfr.201000108

[CR204] Wohlmuth H, Leach DN, Smith MK, Myers SP (2005) Gingerol content of diploid and tetraploid clones of ginger (Zingiber officinale roscoe). J Agric Food Chem 53:5772–5778 15998147 10.1021/jf050435b

[CR205] Wu X, Wei J, Yi Y, Gong Q, Gao J (2022) Activation of Nrf2 signaling: a key molecular mechanism of protection against cardiovascular diseases by natural products. Front Pharmacol 13:1057918 36569290 10.3389/fphar.2022.1057918 PMC9772885

[CR206] Wu Y, Sun X, Li H, Chu X, Xue Y, Qi J, Jia Q, Han X, Chu L, Guan S, Wang X (2023) 6-Gingerol attenuates arsenic trioxide-induced liver injury by inhibiting pyroptosis and ROS-NLRP3 inflammatory signaling pathway: based on network pharmacology analysis and experiment verification. J Funct Foods 105:105551

[CR207] Xia C, Dong R, Chen C, Wang H, Wang DW (2015) Cardiomyocyte specific expression of acyl-coA thioesterase 1 attenuates sepsis-induced cardiac dysfunction and mortality. Biochem Biophys Res Commun 468:533–540 26518651 10.1016/j.bbrc.2015.10.078

[CR208] Xiang S, Jian Q, Chen W, Xu Q, Li J, Wang C, Wang R, Zhang D, Lin J, Zheng C (2024) Pharmacodynamic components and mechanisms of ginger *(Zingiber officinale*) in the prevention and treatment of colorectal cancer. J Ethnopharmacol 324:117733 38218504 10.1016/j.jep.2024.117733

[CR209] Xie X, Sun S, Zhong W, Soromou LW, Zhou X, Wei W, Ren Y, Ding Y (2014) Zingerone attenuates lipopolysaccharide-induced acute lung injury in mice. Int Immunopharmacol 19:103–109 24412620 10.1016/j.intimp.2013.12.028

[CR210] Young HY, Luo YL, Cheng HY, Hsieh WC, Liao JC, Peng WH (2005) Analgesic and anti-inflammatory activities of [6]-gingerol. J Ethnopharmacol 96(1–2):207–210 15588672 10.1016/j.jep.2004.09.009

[CR211] Yu W, Tu Y, Long Z, Liu J, Kong D, Peng J, Wu H, Zheng G, Zhao J, Chen Y, Liu R (2022a) Reactive oxygen species bridge the gap between chronic inflammation and tumor development. Oxid Med Cell Longev 2022(1):2606928 35799889 10.1155/2022/2606928 PMC9256443

[CR212] Yu Y, Guo D, Zhao L (2022b) MiR-199 aggravates doxorubicin-induced cardiotoxicity by targeting TAF9b. Evid-Based Complement Altern Med 2022:4364779 10.1155/2022/4364779 PMC9307339 35873641

[CR213] Yücel Ç, Karatoprak GŞ, Açıkara ÖB, Akkol EK, Barak TH, Sobarzo-Sánchez E, Aschner M, Shirooie S (2022) Immunomodulatory and anti-inflammatory therapeutic potential of gingerols and their nanoformulations. Front Pharmacol 13:902551 36133811 10.3389/fphar.2022.902551 PMC9483099

[CR214] Zadorozhna M, Mangieri D (2021) Mechanisms of chemopreventive and therapeutic proprieties of ginger extracts in cancer. Int J Mol Sci 22(12):6599 34202966 10.3390/ijms22126599 PMC8234951

[CR215] Zappavigna S, Cossu AM, Grimaldi A, Bocchetti M, Ferraro GA, Nicoletti GF, Filosa R, Caraglia M (2020) Anti-inflammatory drugs as anticancer agents. Int J Mol Sci 21(7):2605 32283655 10.3390/ijms21072605 PMC7177823

[CR216] Zhang F, Zhang J, Qu J, Zhang Q, Prasad C, Wei Z (2017) Assessment of anti-cancerous potential of 6-gingerol (Tongling White Ginger) and its synergy with drugs on human cervical adenocarcinoma cells. Food Chem Toxicol 109(Pt 2):910–922 10.1016/j.fct.2017.02.038 28249781

[CR217] Zhang F, Zhang J, Yang W, Xu P, Xiao Y, Zhang H (2018a) 6-Gingerol attenuates LPS-induced neuroinflammation and cognitive impairment partially via suppressing astrocyte overactivation. Biomed Pharmacother 107:1523–1529 30257370 10.1016/j.biopha.2018.08.136

[CR218] Zhang Q-Y, Wang F-X, Jia K-K, Kong L-D (2018b) Natural product interventions for chemotherapy and radiotherapy-induced side effects. Front Pharmacol 9:1253. 10.3389/fphar.2018.01253 30459615 10.3389/fphar.2018.01253 PMC6232953

[CR219] Zhang X, Hu C, Kong CY, Song P, Wu HM, Xu SC, Yuan YP, Deng W, Ma ZG, Tang QZ (2020) FNDC5 alleviates oxidative stress and cardiomyocyte apoptosis in doxorubicin-induced cardiotoxicity via activating AKT. Cell Death Differ 27(2):540–555 31209361 10.1038/s41418-019-0372-z PMC7206111

[CR220] Zhang X, Li X, Xia R, Zhang H-S (2024) Ferroptosis resistance in cancer: recent advances and future perspectives. Biochem Pharmacol 219:115933 37995980 10.1016/j.bcp.2023.115933

[CR221] Zhao H, Eguchi S, Alam A, Ma D (2017) The role of nuclear factor-erythroid 2 related factor 2 (Nrf-2) in the protection against lung injury. Am J Physiol-Lung Cell Mol Physiol 312(2):L155–L162 27864288 10.1152/ajplung.00449.2016

[CR222] Zhao L, Zhou X, Xie F, Zhang L, Yan H, Huang J, Zhang C, Zhou F, Chen J, Zhang L (2022) Ferroptosis in cancer and cancer immunotherapy. Cancer Commun 42(2):88–116 10.1002/cac2.12250 PMC8822596 35133083

[CR223] Zhao D, Yang K, Guo H, Zeng H, Wang S, Xu H, Ge A, Zeng L, Chen S, Ge J (2023) Mechanisms of ferroptosis in Alzheimer’s disease and therapeutic effects of natural plant products: a review. Biomed Pharmacother 164:114312 37210894 10.1016/j.biopha.2023.114312

[CR224] Zhou L, Yu P, Wang T, Du Y, Chen Y, Li Z, He M, Feng L, Li H, Han X, Ma H, Liu H (2022) Polydatin attenuates cisplatin-induced acute kidney injury by inhibiting ferroptosis. Oxid Med Cell Longev. 10.1155/2022/9947191 35075382 10.1155/2022/9947191 PMC8783728

[CR225] Zhou P, Wang Z, Chen C, Famurewa AC, Olatunji OJ (2023) Naringin ameliorates 5-fluorouracil elicited neurotoxicity by curtailing oxidative stress and iNOS/NF-ĸB/caspase-3 pathway. Open Chem 21:20230126

[CR226] Zhu H, Sarkar S, Scott L, Danelisen I, Trush MA, Jia Z, Li YR (2016) Doxorubicin redox biology: redox cycling, topoisomerase inhibition, and oxidative stress. Reactive Oxygen Species (Apex, NC) 1(3):189 10.20455/ros.2016.835 PMC5921833 29707645

[CR227] Zirak MR, Karimi G, Rahimian R, Jafarian AH, Hayes AW, Mehri S (2020) Tropisetron ameliorates cyclophosphamide-induced hemorrhagic cystitis in rats. Eur J Pharmacol 883:173310 32619674 10.1016/j.ejphar.2020.173310

